# Comparative Analysis of the Development of Acquired Radioresistance in Canine and Human Mammary Cancer Cell Lines

**DOI:** 10.3389/fvets.2020.00439

**Published:** 2020-07-23

**Authors:** Mark Gray, Arran K. Turnbull, James Meehan, Carlos Martínez-Pérez, Charlene Kay, Lisa Y. Pang, David J. Argyle

**Affiliations:** ^1^The Royal (Dick) School of Veterinary Studies and Roslin Institute, University of Edinburgh, Edinburgh, United Kingdom; ^2^Translational Oncology Research Group, Institute of Genetics and Molecular Medicine, Western General Hospital, University of Edinburgh, Edinburgh, United Kingdom; ^3^Breast Cancer Now Edinburgh Research Team, Institute of Genetics and Molecular Medicine, Western General Hospital, University of Edinburgh, Edinburgh, United Kingdom

**Keywords:** canine breast cancer models, human breast cancer, radioresistance, global gene analysis, characterization of radioresistant cell lines, comparative oncology

## Abstract

Research using *in vitro* canine mammary cancer cell lines and naturally-occurring canine mammary tumors are not only fundamental models used to advance the understanding of cancer in veterinary patients, but are also regarded as excellent translational models of human breast cancer. Human breast cancer is commonly treated with radiotherapy; however, tumor response depends on both innate radiosensitivity and on tumor repopulation by cells that develop radioresistance. Comparative canine and human studies investigating the mechanisms of radioresistance may lead to novel cancer treatments that benefit both species. In this study, we developed a canine mammary cancer (REM-134) radioresistant (RR) cell line and investigated the cellular mechanisms related to the development of acquired radioresistance. We performed a comparative analysis of this resistant model with our previously developed human breast cancer radioresistant cell lines (MCF-7 RR, ZR-751 RR, and MDA-MB-231 RR), characterizing inherent differences through genetic, molecular, and cell biology approaches. RR cells demonstrated enhanced invasion/migration capabilities, with phenotypic evidence suggestive of epithelial-to-mesenchymal transition. Similarities were identified between the REM-134 RR, MCF-7 RR, and ZR-751 RR cell lines in relation to the pattern of expression of both epithelial and mesenchymal genes, in addition to WNT, PI3K, and MAPK pathway activation. Following the development of radioresistance, transcriptomic data indicated that parental MCF-7 and ZR-751 cell lines changed from a luminal A classification to basal/HER2-overexpressing (MCF-7 RR) and normal-like/HER2-overexpressing (ZR-751 RR). These radioresistant subtypes were similar to the REM-134 and REM-134 RR cell lines, which were classified as HER2-overexpressing. To our knowledge, our study is the first to generate a canine mammary cancer RR cell line model and provide a comparative genetic and phenotypic analysis of the mechanisms of acquired radioresistance between canine and human cancer cell lines. We demonstrate that the cellular processes that occur with the development of acquired radioresistance are similar between the human and canine cell lines; our results therefore suggest that the canine model is appropriate to study both human and canine radioresistant mammary cancers, and that treatment strategies used in human medicine may also be applicable to veterinary patients.

## Introduction

Naturally-occurring mammary tumors are the most frequently diagnosed cancer in bitches. These neoplasms represent ~50% of all canine tumors (1), of which 50% are malignant (2–4). Due to similarities in clinical features, relative age of onset, risk factors and tumor biology, canine mammary tumors (CMT) represent an excellent comparative and translational model for human breast cancer (HBC) (5–7). Gene expression profiles of primary (8) and metastatic (9) CMT have also shown similarities with HBC profiles, providing evidence that canine models can be utilized to help understand the genetic mechanisms of carcinogenesis in humans and dogs (4, 10, 11).

HBC is routinely managed with surgery followed by combinations of adjuvant chemotherapy, radiotherapy (RT), endocrine therapy or targeted therapy. RT is a commonly used breast cancer treatment; estimates indicate that curative or palliative RT can benefit ~83% of breast cancer patients (12). The use of adjuvant whole-breast RT after breast-conserving surgery has also been shown to deliver regional disease control and overall survival rates comparable with patients receiving a mastectomy, while also reducing side effects and improving cosmetic outcomes (13–15). Although 5-year survival rates for breast cancer patients following RT are ~80%, 30% will subsequently develop local recurrence and/or metastasis. Unfortunately, the vast majority of these patients have a poor prognosis and die within 5-years of disease progression (16). Cancer cells that possess intrinsic radioresistance, or develop acquired resistance during RT, can repopulate the tumor site after treatment. This can lead to treatment failures with the development of tumor recurrence and/or metastatic disease. Multiple factors are associated with the ability of cancer cells to develop acquired radioresistance, including signaling pathway dysregulation (e.g., EGFR/PI3K/AKT/mTOR), activation of DNA damage repair mechanisms, the existence of cancer stem cells, alterations in cancer metabolism and epithelial-to-mesenchymal transition (EMT). Hypoxic tumor microenvironments can also drive cancer cells to adopt an aggressive, treatment resistant phenotype (17). In order to develop treatment strategies to overcome/target the clinical issue of radioresistance, we require a detailed understanding of the mechanisms underlying acquired radioresistance.

In HBC, accurate disease staging is a mandatory requirement before beginning definitive treatment; classification systems that provide both predictive and prognostic information are commonly used to inform patient treatment (18). Histological grading with assessment of human epidermal growth factor 2 (HER2), estrogen receptor (ER), and progesterone receptor (PR) status provides an indication of the drivers of the disease and influences endocrine and/or targeted therapy use (19, 20). HBC can also be classified into intrinsic molecular subtypes that can predict treatment responses, overall survival and disease-free survival (21–24). These subtypes include: normal breast-like, luminal A, luminal B, HER2^+^, basal and “claudin-low” (21, 22, 24, 25). CMT can be solely epithelial (simple carcinoma or adenoma) or mesenchymal (fibrosarcoma, fibroadenoma, sarcoma, or osteosarcoma) in origin; however, a combination of mesenchymal and epithelial (carcinosarcoma or benign mixed tumors) or myoepithelial and epithelial tissues (complex adenoma or complex carcinoma) can also occur (26). Canine inflammatory mammary carcinoma is a rare CMT subtype that carries a very poor prognosis (27, 28). In contrast to human medicine, the diagnosis of CMT relies mainly on this histological grading system without undergoing specific receptor status evaluation or molecular subtype classification. The lack of integration of these diagnostic tests into the treatment decision-making process is largely because surgery alone is the main treatment option for dogs.

The effects of estrogen on CMT development have been previously established from a study that assessed the incidence of CMT in spayed and intact bitches. This study showed that only 0.5% of dogs spayed prior to their first season developed mammary tumors. However, levels rose to 8% and 26% when bitches were neutered following their first or second seasons, respectively. Additionally, no preventative effect was observed on the risk of CMT development when bitches were spayed following their second season (29). Although estrogen is associated with CMT development, the utilization of endocrine therapies, such as the selective ER modulator tamoxifen, has been reported in the literature only a limited number of times (30, 31). Conflicting results produced from these studies, combined with severe side effects associated with the use of anti-oestrogens in dogs (vulvar oedema, vaginal discharge, pyometra, and retinitis), have restricted their use in the treatment of CMT (31, 32). RT is infrequently employed in CMT treatment but could be used to improve regional disease control when cancer-free margins cannot be obtained at surgery, or as a palliative therapy for inflammatory mammary carcinomas or non-resectable tumors. Additional studies to elucidate the future role of RT in CMT treatment regimens are needed due to the impact it has in the management of HBC patients (33).

Compared to chemoresistance, there is an inadequate understanding of the mechanisms underlying radioresistance; this partly results from a lack of model systems for both human and veterinary applications. To begin to address this issue we have recently developed and characterized 3 novel HBC radioresistant (RR) cell lines (34). These included hormone-dependent ER^+^/PR^+^/HER2^−^ (MCF-7 and ZR-751) and hormone-independent ER^−^/PR^−^/HER2^−^ (MDA-MB-231) cell lines that represented different molecular HBC subtypes. In this new study we now go on to develop a novel CMT radioresistant cell line (REM-134), investigate the mechanisms of CMT radioresistance and perform a comparative analysis with our HBC RR cell line models. Although multiple CMT cell lines have previously been generated (35–38), the REM-134 cell line was the first to be developed, derived from a canine mammary carcinoma (39), a commonly occurring disease subtype with a poor prognosis. Even though it is commonly used in research, we chose to use the REM-134 cell line as it has yet to be fully characterized, especially in terms of its hormone receptor status. Its use in this study would therefore provide valuable information. Through genotypic, phenotypic, and functional analysis of our developed RR cell lines, we provide evidence that the cellular processes that occur with the development of acquired radioresistance are similar between human and canine cell lines; this suggests that not only is our canine model appropriate to study both canine and human radioresistant mammary cancers, but that treatment strategies used in human medicine may also be applicable to veterinary patients. To our knowledge, our study is the first to generate a CMT radioresistant model and provide a comparative analysis of the mechanisms of acquired radioresistance between human and canine breast cancer cell lines. We are also the first to report the use of canine multicellular tumor spheroids (MTS) originating from RR cells in immunohistochemical analysis and functional assays.

## Materials and Methods

### Cell Culture

Unless otherwise stated, reagents used for cell culture were acquired from Gibco Thermo Fisher Scientific (Paisley, UK). The HBC cell lines MCF-7, ZR-751 and MDA-MB-231 were cultured using Dulbecco's modified Eagle's medium (DMEM) with 10% fetal calf serum, 50 U ml^−1^ penicillin and 50 mg ml^−1^ streptomycin. The CMT cell line REM-134 was cultured in DMEM (high glucose) with the same additions. All cells were incubated at 37°C in a humidified atmosphere with 5% CO_2_. The HBC cell lines were obtained from the American Type Culture Collection (LGC Standards, Teddington, UK); the REM-134 cell line was a kind gift from Professor R.W. Else (College of Veterinary Medicine, University of Edinburgh, UK). All cell lines were authenticated at Health England (Porton Down, Salisbury, UK) by short tandem repeat (STR) profiling. All experiments were performed using cell lines maintained at low passage numbers with new cells acquired from frozen stocks after 10 passages.

### Irradiation of Cells and Development of Radioresistant Cell Lines

A Faxitron cabinet X-ray system 43855D (Faxitron X-ray Corporation, IL, USA) was used to irradiate cells. Radioresistant cells were established from parental cell lines through exposure to weekly doses of radiation. Beginning with an initial dose of 2 Gy, cells were irradiated weekly with incremental doses of 0.5 Gy for 12-weeks. After this development period cells were maintained with additional doses of 5 Gy given every week. Cells were routinely passaged 24 h after each radiation dose.

### Sulforhodamine B Proliferation (SRB) Assay

Cells were seeded into 96 well plates (500 cells/well). After 24 h of incubation, cells were irradiated and then fixed up to 120 h post-treatment by adding 50 μl 25% trichloracetic acid (Sigma-Aldrich, UK) at 4°C for 1 h. Wells were washed in dH_2_O and dried. Fifty microliter SRB dye [0.4% SRB dissolved in 1% glacial acetic acid (VWR International)] was added to the wells and the cells were incubated for 30 min. The cells were then washed 4 times in 1% glacial acetic acid and incubated for 60 min after the addition of 150 μl 10 mM Tris-NaOH buffer (pH 10.5). A Biohit BP800 spectrophotometer (Biohit Ltd, UK) and Wallac 1,420 Manager program (PerkinElmer, UK) were used to analyze optical density at 540 nm.

### Colony Formation Assay

Cells were irradiated with varying doses 24 h after seeding into 75 mm plates (1,000 cells/plate). 1,9-dimethyl-methylene blue zinc chloride double salt (Sigma-Aldrich, UK) was used to fix and stain the cells between 10 and 14 days after seeding, when colonies consisting of at least 50 cells/colony had formed in the untreated control group. The plating efficiencies and survival fractions for treatment and control colony formation (CF) groups were calculated (40).

### Scratch (Migratory) Assays

Cells were seeded into 6 well plates at densities that led to 100% confluence after 24 h. Scratch assays were carried out as previously described (41) following replacement of the media routinely used for cell culture with 0.1% serum-supplemented DMEM. Phase contrast images were taken (Axiovert DS100, x5 objective) up to 48 h post-scratch. The area lacking migrating cells, expressed as a % of the initial scratched area, was calculated at each time point using FIJI software.

### Formation of Multicellular Tumor Spheroids

A single cell suspension of ~15,000,000 cells was placed into a spinner flask (Cellcontrol Spinner Flask, Integra, Switzerland) containing 100 ml of standard DMEM. The flask was placed onto a magnetic stirrer platform (Cellspin, Integra, Switzerland) with MTS forming over a period of 7 days under routine incubation conditions. Hypoxyprobe-1 (Hypoxyprobe, USA) was used to detect hypoxic areas within MTS. MTS were incubated with 100 μM hypoxyprobe-1 for 1 h before fixation.

### 3D Invasion Assay Using Multicellular Tumor Spheroids

MTS were transferred into the wells of a 24-well plate with 500 μl of collagen mix (0.22 M NaOH (Sigma-Aldrich, UK), fetal calf serum, 10x DMEM (Sigma-Aldrich, UK), cell matrix type 1-A (Alphalabs) and ice cold 0.1% filtered acetic acid at concentrations of 10, 10, 10, 25, and 45% respectively). The plates were incubated at 37°C for 1 h to allow collagen polymerization, after which 500 μl of routine DMEM was added. Phase-contrast images were taken at 24 h time-points up to 96 h (Axiovert DS100, x5 objective). Invasion was measured at each time point with a FIJI macro developed by Matthew Pearson (IGMM Advanced Imaging Resource, University of Edinburgh), with invasion expressed as a % of the initial MTS area.

### Protein Isolation and Detection

Whole cell lysates were prepared as described previously (42). Equal protein amounts were separated by electrophoresis using sodium dodecyl sulfate (SDS) polyacrylamide gels. Proteins were transferred to Immobilon-P transfer membranes (Millipore, UK). Membranes were blocked using Odyssey Blocking Buffer (LI-COR Biosciences, UK) diluted 1:1 with PBS for 1 h. Membranes were then incubated overnight at 4°C with the appropriate primary antibodies (Table 1). IRDye 680LT (Li-Cor 926-68021, 1:10,000) and IRDye 800CW (Li-Cor, 926-32210, 1:10,000) secondary antibodies were added, and signals detected using a Li-Cor Odyssey Imager.

**Table 1:** Primary antibodies used for western blotting (WB), immunocytochemistry (ICC), and immunohistochemistry (IHC).

**Primary antibody target antigen**	**Antibody details**	**Dilutions**	**Antigen retrieval**
Anti-ERα	Mouse mAb; Dako; M7047	1:50 (ICC, IHC)	Sodium citrate
Anti-HER2	Rabbit mAb; Cell signaling technology; 2,242	1:50 (ICC, IHC)	Sodium citrate
Anti-PR	Mouse mAb; Dako; M3569	1:150 (ICC, IHC)	EDTA
Anti-AKT	Mouse mAb; Cell signaling technology; 2,920	1:1000 (WB)	N/A
Anti-Phospho AKT	Rabbit pAb; Cell signaling technology; 9,271	1:1000 (WB)	N/A
Anti-ERK	Rabbit pAb; Cell signaling Technology; 9,102	1:1000 (WB)	N/A
Anti-Phospho ERK	Mouse mAb; Cell signaling technology; 9,106	1:1000 (WB)	N/A
Anti-ki67	Rabbit mAb; Abcam; 92,742	1:150 (ICC, IHC)	Sodium citrate
Anti-hypoxyprobe-1	Mouse mAb; Hypoxyprobe; HP1-100Kit	1:2000 (IHC)	Sodium citrate
Anti-E-cadherin	Mouse mAb; BD transduction; 610,182	1:50 (ICC, IHC)	Sodium citrate
Anti-N-cadherin	Mouse mAb; BD transduction; 610,921	1:150 (ICC, IHC)	Sodium citrate
Anti-vimentin	Mouse mAb; Abcam; 8,069	1:50 (ICC, IHC)	Sodium citrate
Anti-SNAIL	Rabbit pAb; Abcam; 128,530	1:250 (ICC, IHC)	Sodium citrate
Anti-WNT5a	Mouse mAb; Thermo Scientific; MA5-15,502	1:500 (IHC)	Sodium citrate
Anti-Frizzled	Mouse pAb; R&D Systems; AF1120	1:50 (IHC)	Sodium citrate

### Immunohistochemistry

MTS were fixed in 4% formaldehyde (Genta Medical, UK) for 24 h. After fixation, the MTS were placed in 2% agarose and processed using the Thermo Scientific Excelsior AS Tissue Processor (Thermo Scientific, UK) and embedded in paraffin. Blocks containing MTS were cut with a Leica RM2235 rotary microtome (Leica Microsystems Ltd, UK); 4 μm sections were placed on SuperFrost Plus glass slides (Thermo Scientific, UK) and dried for 4 h at 53°C.

MTS were deparaffinized and rehydrated before undergoing antigen retrieval (Table 1). Incubation with 3% H_2_O_2_ solution (Dako, UK) was performed for 10 min to block endogenous peroxidase activity. Total Protein Block (Dako, UK) was used for 10 min to block non-specific antibody staining with the MTS subsequently incubated with primary antibodies for 1 h (Table 1). Envision labeled polymer (Dako, UK) was added for 30 min, after which DAB and substrate buffer (1:50) (Dako, UK) were added to visualize protein staining. Haematoxylin was used to counterstain the MTS, which were then dehydrated and mounted with coverslips using DXP mountant (Sigma-Aldrich, UK). A NanoZoomer ER slide scanner (Hamamatsu Photonics, UK) was used to scan all slides. The stained MTS were viewed using NanoZoomer Digital Pathology software. Image analysis was performed using QuPath version 0.1.2 (43).

### RNA Extraction and Whole-Transcriptome Gene Expression Analysis

Cells were seeded in triplicate into 75 mm plates (3,000,000 cells/plate). After 24 h of incubation, pellets containing up to 10,000,000 cells were collected by trypsinisation, snap-frozen on dry ice and stored at −70°C for RNA extraction. The RNeasy Mini Kit using QIAshredder technology (UK Qiagen, Ltd) was used to extract RNA from the cells. Total RNA was purified from animal cells using spin technology, as per the manufacturer's protocol. The NanoDropTM Spectrophotometer ND1000 (Thermo Fischer Scientific) was used to quantify the RNA and assess for contaminants. RNA quality was evaluated by producing an RNA integrity number (RIN) for each sample (Agilent Bioanalyzer); each of the samples had RIN values above 9.7 (Supplementary Table 1). Lexogen QuantSeq 3' FWD sequencing technology was used to produce full genome expression read-counts on an Illumina flow cell; this was scanned with the Illumina HiScanSQ system (Edinburgh Clinical Research Facility's Genetic Core, University of Edinburgh). Next generation sequencing reads were generated toward the poly(A) tail with read 1 directly reflecting the mRNA sequence. The BlueBee high-performance next generation sequencing analysis software was used to pre-process the FASTQ files; this implements poly(A) tail trimming and alignment to the Genome Reference Consortium Human genome build 38 reference genome (for human cell lines) and the *Canis lupus familiaris* reference genome (for canine cell lines) using the Spliced Transcripts Alignment to a Reference (STAR) algorithm (44).

Data were filtered, removing all genes with <5 reads per sample in at least 90% of samples. Cell lines were mapped to species-specific Ensembl gene identifiers and cross-species matches were determined using Ensembl BioMART (45). In total 17,243 genes were mapped to human Ensembl gene identifiers and 13,703 were mapped to canine Ensembl gene identifiers. Following cross-species mapping, 9,692 genes were identified as common to both datasets. No significant difference in variance of expression was observed across all genes between both datasets (Supplementary Figure 1). Before the analysis took place, data were log2 transformed and quantile normalized in R (Bioconductor) software and packages (46). Heatmap and cluster analysis were implemented with the TM4 MeV (multiple experiment viewer) software (47). Heatmap clustering was performed using Pearson correlation with average linkage. Correction for batch effects was performed to integrate gene expression data produced in this study with public datasets; this was achieved using R with the ComBat package, as described previously (48, 49). Gene enrichment analysis was performed using the DAVID Functional Annotation Bioinformatics Microarray Analysis tool (50). Hierarchical clustering of both the parental and RR cell lines was accomplished using a published list of genes; the expression profile of the genes within this list denotes the breast cancer intrinsic subtypes (luminal A, luminal B, normal-like, basal, and HER2) (24). The *genefu* R package (51) was used to assign samples to the differing intrinsic subtypes. This package applies a Single Sample Predictor (SSP) algorithm which is a nearest-centroid classifier. Centroids signifying the molecular subtypes of breast cancer were identified through hierarchical clustering using the same intrinsic gene list that was used for cluster analysis within this study. All datasets generated and/or analyzed within this study are available in the NCBI's Gene Expression Omnibus (52) and are accessible through GEO Series accession number GSE149988.

### Statistical Analysis

Two-way ANOVA with Holm-Šídák multiple comparisons test was employed in SRB, CF, and migration/invasion assays. Unpaired (two tailed) *t-*test was employed in the IHC analysis. *P* < 0.05 were deemed statistically significant. Data are shown as mean ± SEM. Statistical analysis was performed and graphs were generated with GraphPad Prism 8.

## Results

### Development and Confirmation of Acquired Radioresistance in Canine and Human Breast Cancer Cell Lines

**Figure 1:**
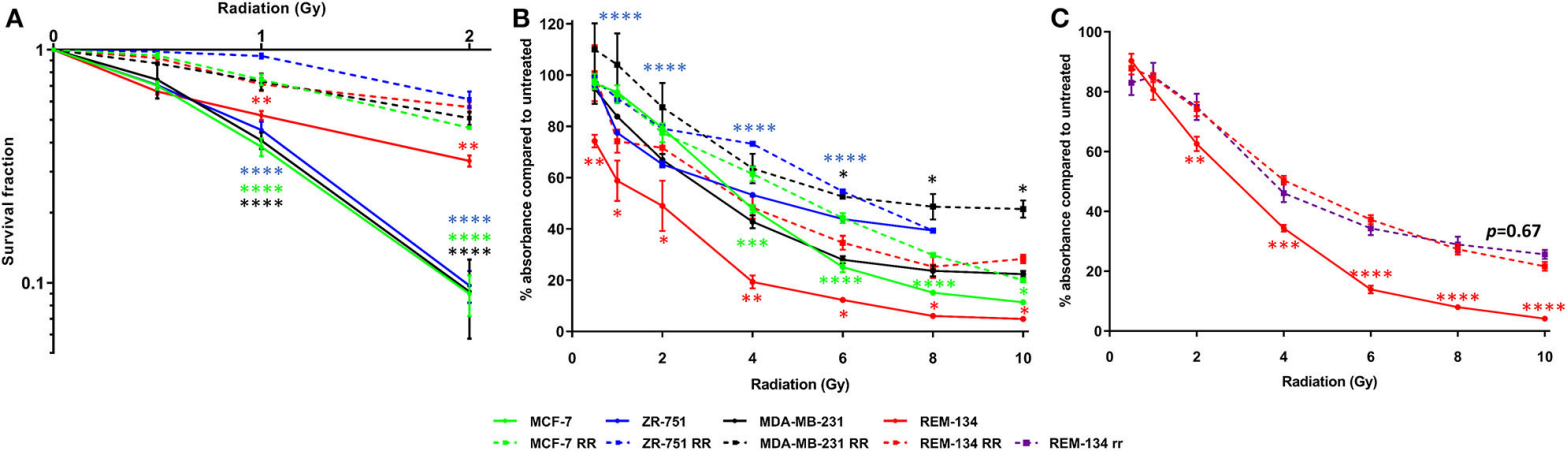
Confirmation of radioresistance using colony formation and SRB assays. **(A)** Colony formation assays comparing MCF-7, ZR-751, MDA-MB-231, and REM-134 cell lines with their derived RR cell lines at 10–14 days post-radiation treatment. **(B)** SRB assays comparing MCF-7, ZR-751, MDA-MB-231, and REM-134 cell lines with their derived RR cell lines at 120 h post-radiation treatment. **(C)** SRB assays comparing REM-134, REM-134 RR, and REM-134 rr cell lines at 120 h post-radiation treatment. The REM-134 rr is a radioresistant cell line that had not been radiated for 6 months (24 passages) before the experiment (2-way ANOVA with Holm-Šídák multiple comparisons test; data expressed as mean ± SEM, *n* = 3, ****p ≤ 0.0001; ***p ≤ 0.001; **p ≤ 0.01; *p ≤ 0.05).

Parental cell line intrinsic radiosensitivity and confirmation of acquired radioresistance in the developed cell lines were investigated through CF and SRB assays. Using the survival fraction of cells that were given a 2 Gy dose of radiation (SF2, a recognized experimental measure of radiosensitivity), a range of intrinsic radiosensitivities was found to be present in the parental cell lines, with the REM-134 cell line showing significantly greater radioresistance compared with the human cell lines. Within the parental human cell lines SF2 was not associated with molecular subtype. The ability of RR cells to form colonies was significantly higher than that of parental cells when subjected to a single radiation dose of up to 2 Gy, confirming the acquisition of radioresistance (Figure 1A). Significantly less inhibition of proliferation was also observed in RR cells in comparison to their parental cells when exposed to radiation doses of up to 10 Gy (Figure 1B). REM-134 RR and REM-134 RR cells that had not been exposed to radiation for 6 months (REM-134 rr) showed similar levels of radioresistance; this suggested that the changes involved in the acquisition of the RR phenotype are maintained over a long period of time (Figure 1C).

### Gene Expression Analysis Identifies Differences Between the Canine Parental and Radioresistant Cell Lines

**Figure 2:**
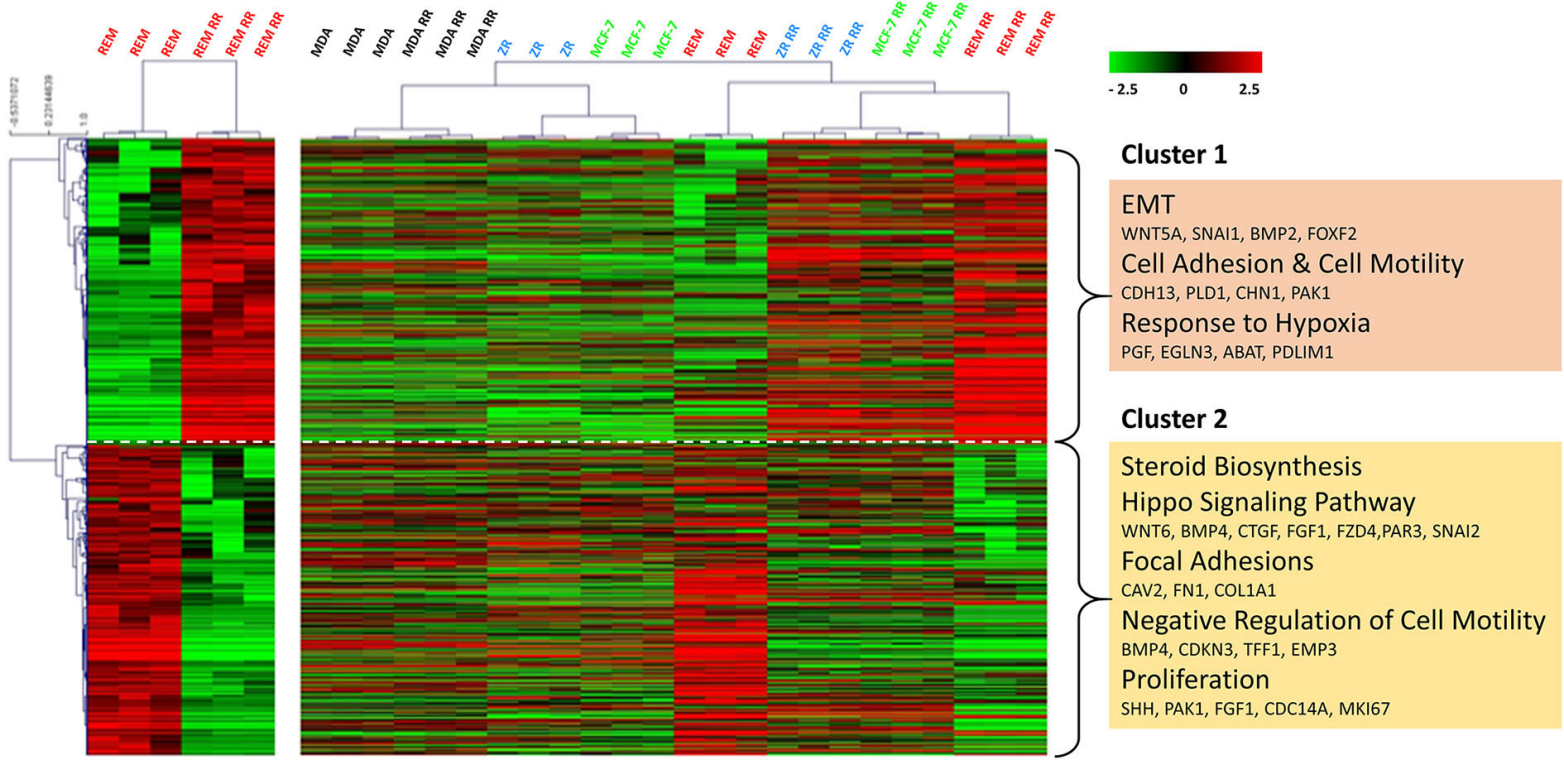
Global gene expression changes between the canine and human parental and RR cell lines. Heatmap showing log2 mean-centered gene expression profiles between parental and RR cell lines in respect of differentially expressed genes (false discovery rate = 0.01). Differences between the REM-134 and REM-134 RR cell lines are shown in the heatmap on the left, and the expression of the same differentially expressed genes across all human and canine parental and RR cells are shown in the heatmap on the right. Heatmap clustering was carried out using Pearson correlation with average linkage; red = higher expression, black = no change, green = lower expression. The gene list and the order in which they appear in the heatmap are shown in Supplementary Table 2.

Using an unsupervised analysis, a large number of genes were found to be inherently differentially expressed (using DESeq2—R, Bioconductor package) between the REM-134 and REM-134 RR cell lines (Figure 2). Higher expression of genes enriched for EMT, cell adhesion/motility, and response to hypoxia (cluster 1), with lower expression of genes involved in steroid biosynthesis, HIPPO signaling, focal adhesion, negative regulators of cell motility and proliferation (cluster 2) were evident in the REM-134 RR cell line compared to its parental cell line. Cluster analysis using these differentially expressed genes was performed using all human and canine parental and RR cell lines. The relative expression of key differentially expressed genes between the REM-134 and REM-134 RR cells associated with EMT (Cluster 1: BMP2, WNT5A, and SNAI1) and HIPPO signaling (Cluster2: WNT6, BMP4, FZD4, SNAI2) across all cell lines are shown in Supplementary Figure 2.

Overall, results showed that the MDA-MB-231, MDA-MB-231 RR, MCF-7, and ZR-751 cell lines clustered together with similar expression patterns across all genes. The MCF-7 RR, ZR-751 RR, REM-134, and REM-134 RR cell lines formed a separate cluster. Interestingly, the MCF-7 RR and ZR-751 RR cells were found to have higher expression of cluster 1 genes compared to their parental lines, undergoing similar radiation-induced changes in expression to the REM-134 cells, with little change in expression of cluster 2 genes. These data suggest a common link between the expression of genes in cluster 1 and the development of radioresistance in the ER^+^ HBC and REM-134 cell lines. Cluster 2 expression appears to be specific to the REM-134 cell line; the expression of these genes does not appear to change in the MDA-MB-231 cells, suggesting that a different mechanism may underlie the development of radioresistance in this triple-negative model (Figure 2 and Supplementary Table 2). Analysis of these global gene changes provided the basis for further investigation of the key enriched pathways associated with the response to radiation.

### Canine Radioresistant Cells Have Lower Expression of Cell Cycle-Associated Genes and Decreased Proliferation

**Figure 3:**
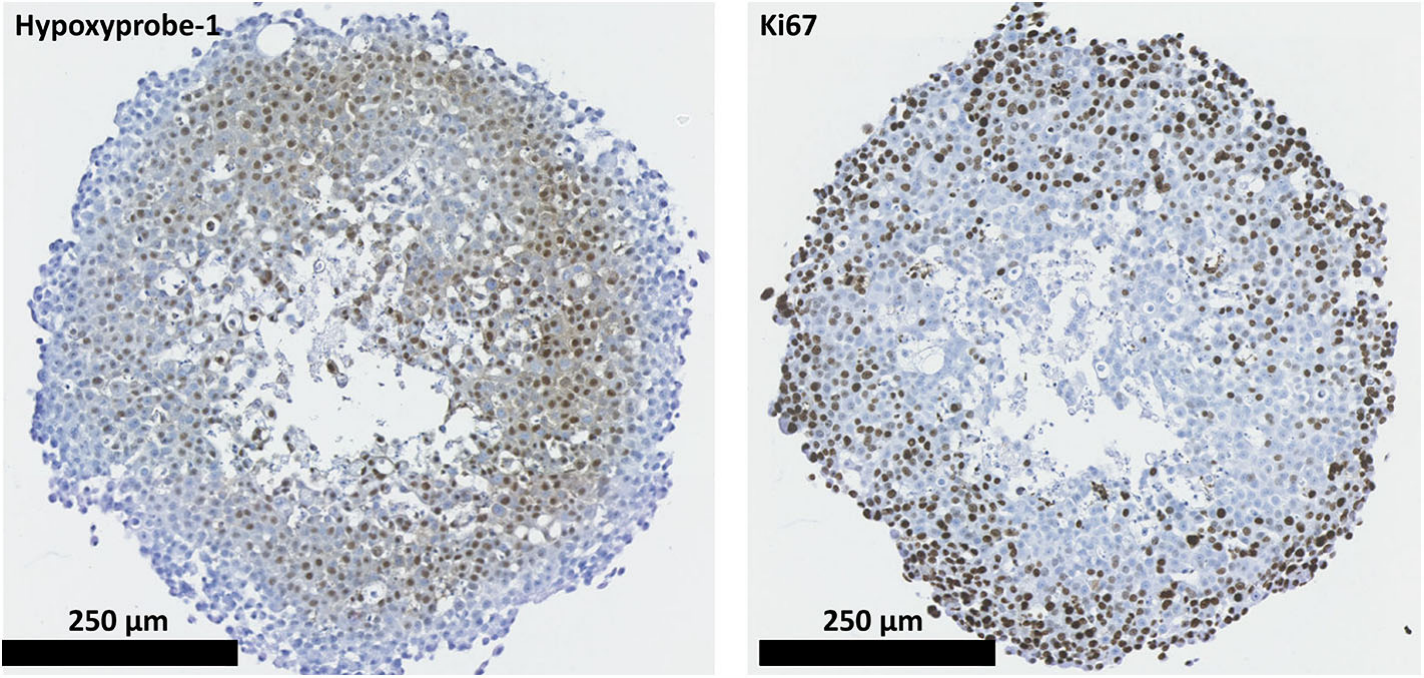
REM-134 MTS show hypoxic and proliferative gradients. The hypoxyprobe-1 compound was used to detect hypoxic regions within REM-134 MTS, while ki67 was used identify proliferating cells.

**Figure 4:**
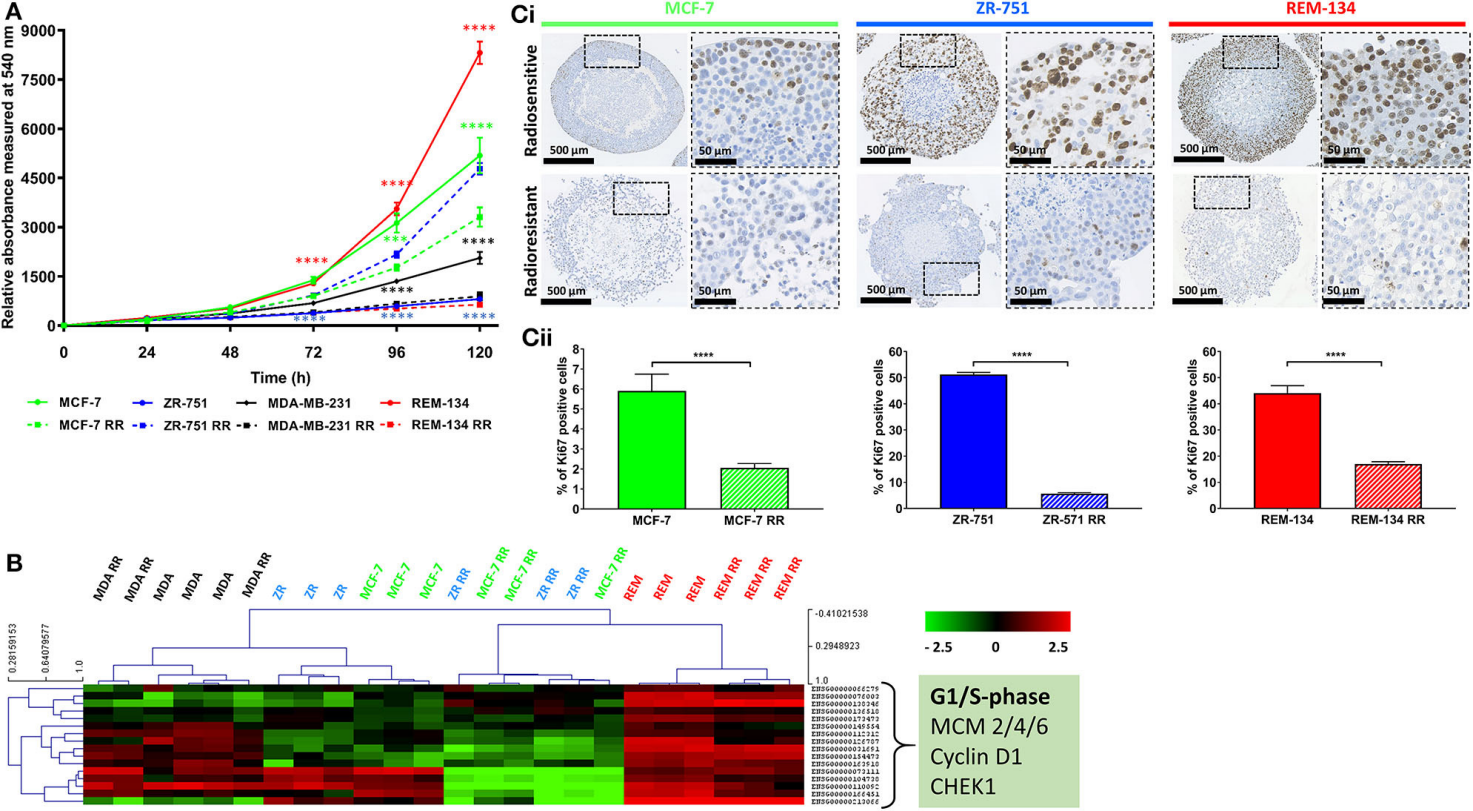
Radioresistant cell lines have modified basal proliferation rates relative to their parental cells. **(A)** SRB assays showing differences in proliferation rates between MCF-7, ZR-751, MDA-MB-231, and REM-134 cell lines and their derived RR cell lines grown in 2D cultures (2-way ANOVA with Holm-Šídák multiple comparisons test; data expressed as mean ± SEM, *n* = 3, ****p ≤ 0.0001; ***p ≤ 0.001). **(B)** Heatmap showing log2 mean-centered gene expression profiles of proliferation genes in parental and RR cell lines showing key G1/S phase regulators taken from the KEGG database cell cycle pathway (55); red = higher expression, black = no change, green = lower expression. Heatmap clustering was carried out using Pearson correlation with average linkage. The gene list is shown in Supplementary Table 3. **(Ci)** IHC of MTS stained for Ki67 using MCF-7, ZR-751, and REM-134 parental and RR cell lines. **(Cii)** Quantitative analysis of the % of cells with Ki67 staining (unpaired, two tailed *t-*test; data expressed as mean ± SEM, *n* = 3, ****p ≤ 0.0001).

2D and 3D cell models were used to assess the proliferative capabilities of parental and RR cells. MTS were the 3D model system used; IHC showed the presence of hypoxic areas both within and surrounding a central necrotic core, with a proliferating layer of cells around the periphery. These are recognized characteristics of MTS; therefore, their presence validated the use of the REM-134 MTS within this study (Figure 3). SRB assays were used to evaluate proliferation rates of cells in 2D cultures (Figure 4A). A range of proliferation rates were found to be present in the parental cell lines, with the REM-134 cell line showing a significantly greater proliferation rate compared with the human cell lines. Comparisons between the parental and RR cell lines identified lower rates of proliferation in MCF-7 RR, MDA-MB-231 RR, and REM-134 RR cells compared to their respective parental cell lines. The opposite was seen for the ZR-751 cell line, where the ZR-751 RR showed higher proliferation rates compared to the parental cell line. Gene expression data showed that both MCF-7 RR and ZR-75-1 RR cell lines exhibited lower expression levels of genes related to cell cycle regulation and G1/S-phase transition, including MCM4, MCM6, and cyclin D1 compared to their parental lines (Figure 4B and Supplementary Table 3). Expression levels of these genes were similar across the parental and RR MDA-MB-231 cell lines, both of which clustered together. In accordance with the SRB results, overall expression levels of these proliferation genes were higher in the REM-134 parental cells compared to all others. Lower expression levels of some genes were observed in the RR compared to the parental REM-134 cells, but overall expression remained higher than in the ER^+^ RR cell lines. Subsequent investigation of proliferation was carried out through IHC; MTS were stained for the proliferation marker Ki67 (Figure 4C) (both parental and RR MDA-MB-231 cells fail to generate MTS that withstood IHC processing). Quantitative IHC analysis identified that there was a lower percentage of Ki67-positive cells in the MCF-7 RR, ZR-751 RR, and REM-134 RR MTS compared with MTS formed from their parental cells. Overall, these results suggested that the RR cells have lower basal proliferation rates compared to their parental cells.

### Canine Radioresistant Cells Have Increased Invasion and Migration Potential

**Figure 5:**
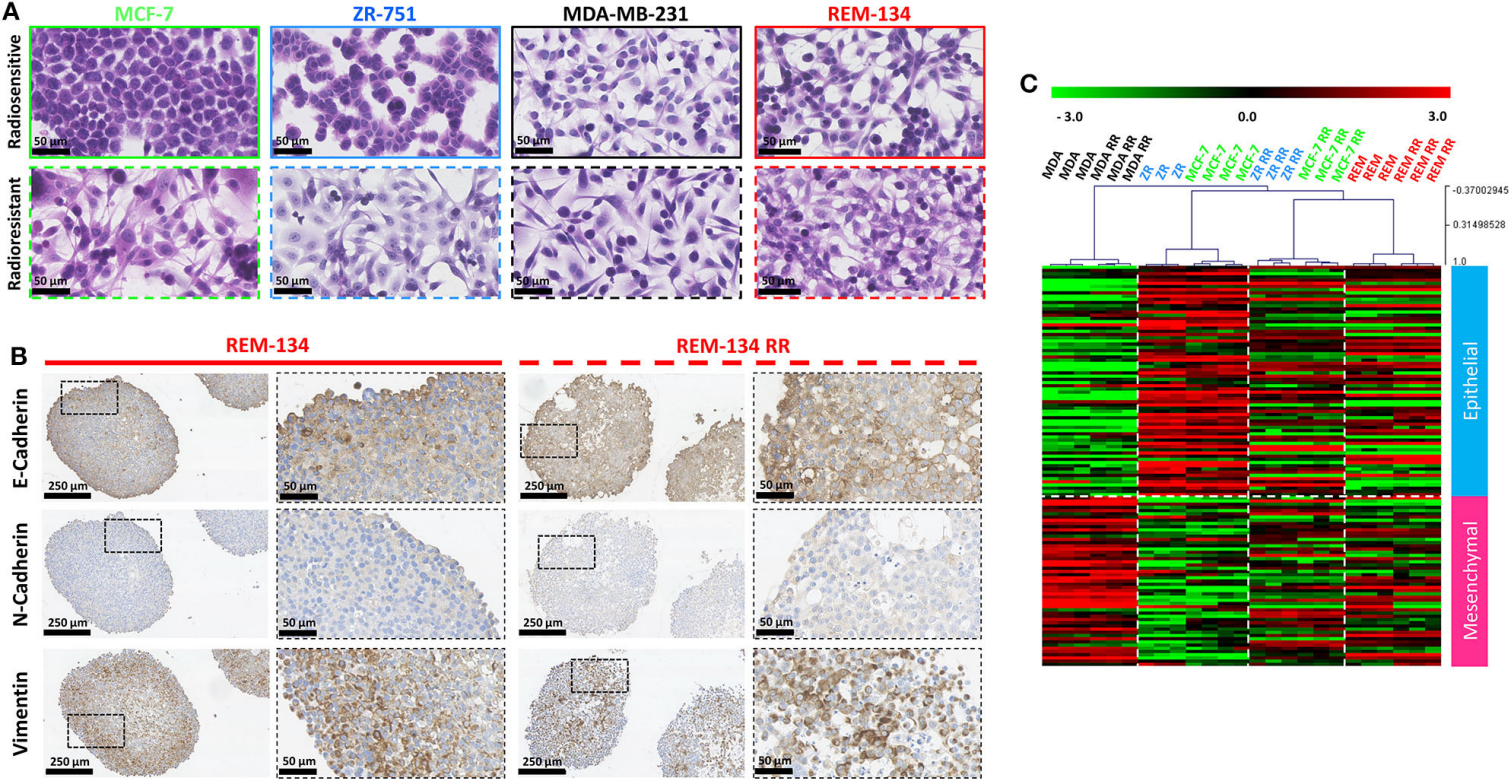
REM-134 and REM-134 RR exhibit signs of EMT, with gene expression profiles similar to the human RR cell lines derived from ER^+^ cells lines. **(A)** H&E staining of cells grown in 2D cultures detailing the morphological differences between parental and RR cell lines. **(B)** IHC staining of EMT markers (vimentin, E-cadherin, and N-cadherin) in REM-134 and REM-134 RR MTS. **(C)** Heatmap showing log2 mean-centered gene expression profiles in respect of a published cancer cell EMT-signature (53); red = higher expression, black = no change, green = lower expression. The gene list and the order in which they appear in the heatmap are shown in Supplementary Table 4.

Morphological changes that occurred with the acquisition of radioresistance were identified through H&E staining of cells grown in 2D cultures. The human parental ER^+^ cell lines (MCF-7 and ZR-751) exhibited an epithelial-like morphology, comprising of tightly packed cells that form cobblestone-like monolayers, typical of luminal subtypes. However, their RR derivatives showed mesenchymal-type characteristics, with cells gaining a spindle-shaped morphology that contacted neighboring cells through focal points rather than the entire cellular circumference. The parental human ER^−^ (MDA-MB-231) and the canine (REM-134) cell lines exhibited a mesenchymal-like phenotype, typical of basal and HER2-overexpressing subtypes; morphological changes in their RR derivatives were not as obvious as those observed in the RR cell lines derived from ER^+^ cells (Figure 5A).

These observed changes in cell morphology provided evidence that the cells were undergoing EMT. We therefore assessed the protein expression levels of EMT markers through IHC. MTS formed from MCF-7 RR and ZR-751 RR cells demonstrated higher expression levels of N-cadherin, vimentin, and SNAIL, in addition to a partial downregulation of E-cadherin. The MDA-MB-231 cell line exhibited high levels of vimentin and SNAIL along with low E-cadherin and N-cadherin expression; no differences between the parental and RR MDA-MB-231 cell lines were identified (Supplementary Figure 3). Although no differences between the REM-134 and REM-134 RR were identified, the pattern of protein expression was similar to that seen in the MCF-7 RR and ZR-751 RR cell lines, with all cell lines showing expression of E-cadherin and vimentin (Figure 5B and Supplementary Figure 3). Gene expression analysis looking at the expression patterns for genes in a published EMT signature produced similar results (53). This study produced a pan-cancer EMT-associated gene expression signature by merging bioinformatic expression data from both The Cancer Cell Line Encyclopedia and The Cancer Genome Atlas (the lists of genes used in our study are provided in Supplementary Table 4). In our analysis, both MCF-7 and ZR-751 parental cell lines exhibited expression patterns consistent with an epithelial genotype, while the parental and RR MDA-MB-231 cells displayed higher expression of mesenchymal genes. The MCF-7 RR, ZR-751 RR, REM-134, and REM-134 RR cell lines all demonstrated a diverse expression pattern, with comparatively higher expression levels of both mesenchymal and epithelial genes, suggestive of a hybrid or transitional phenotype (Figure 5C). The expression levels of key EMT genes (BMP2, WNT5A, and SNAI1) are shown across all cell lines in Supplementary Figure 2. Expression levels of these key genes were found to be increased in the ER^+^ HBC RR and REM-134 RR cell lines compared to their parental lines. A corresponding decrease in expression was observed in key members of the HIPPO tumor suppressor pathway (WNT6, BMP4, FZD4, and SNAI2) in ER^+^ HBC RR and REM-134 RR cell lines compared to their parental lines.

**Figure 6:**
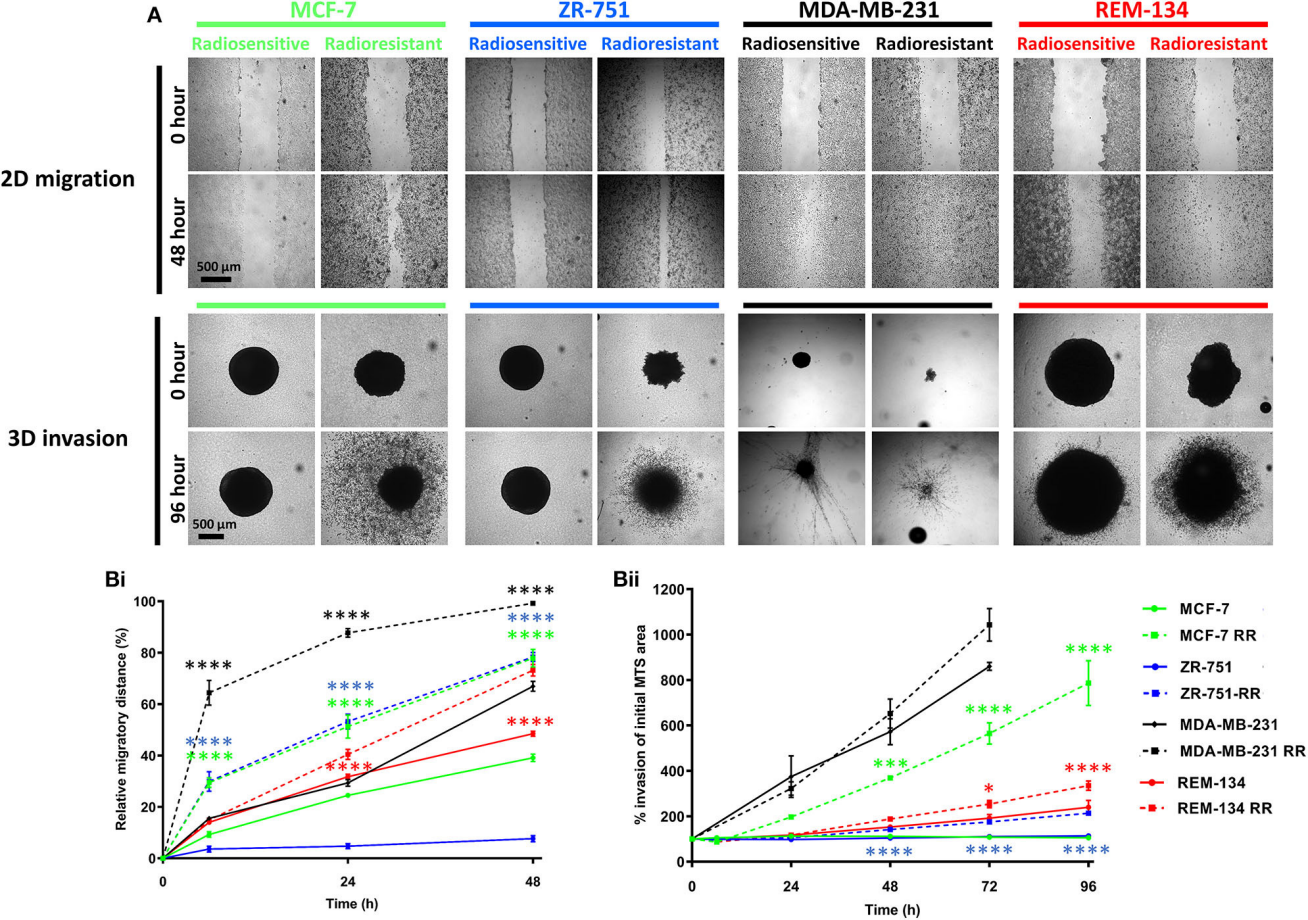
Radioresistant cell lines have increased migration and invasion potential. **(A)** Images of 2D migration and 3D MTS invasion assays comparing the parental and the derived RR cell lines. **(B)** Graphs exhibiting the migration **(Bi)** and invasion assay **(Bii)** results. For the migration assays the relative migratory distance was calculated at each time point up to 48 h and expressed as a % area devoid of cells based on the initial scratched area at day 0. Invasion was assessed up to 96 h post-seeding. Area of MTS at each time point was calculated and expressed as a % of initial MTS area at day 0 (2-way ANOVA with Holm-Šídák multiple comparisons test; data expressed as mean ± SEM, *n* = 3, ****p ≤ 0.0001; ***p ≤ 0.001; *p ≤ 0.05.

Following the identification of cellular changes suggestive of EMT, we investigated the invasive and migratory characteristics of the cell lines. 2D migration assay results demonstrated that the RR cell lines all had significantly increased migratory ability in comparison to their parental cells. Similarly, using 3D invasion assays, the MCF-7 RR, ZR-751 RR, and REM-134 RR cells had increased invasive potential compared to their parental cells (Figures 6A,B).

### Canine Radioresistant Cell Lines Exhibit Enhanced WNT Signaling

**Figure 7:**
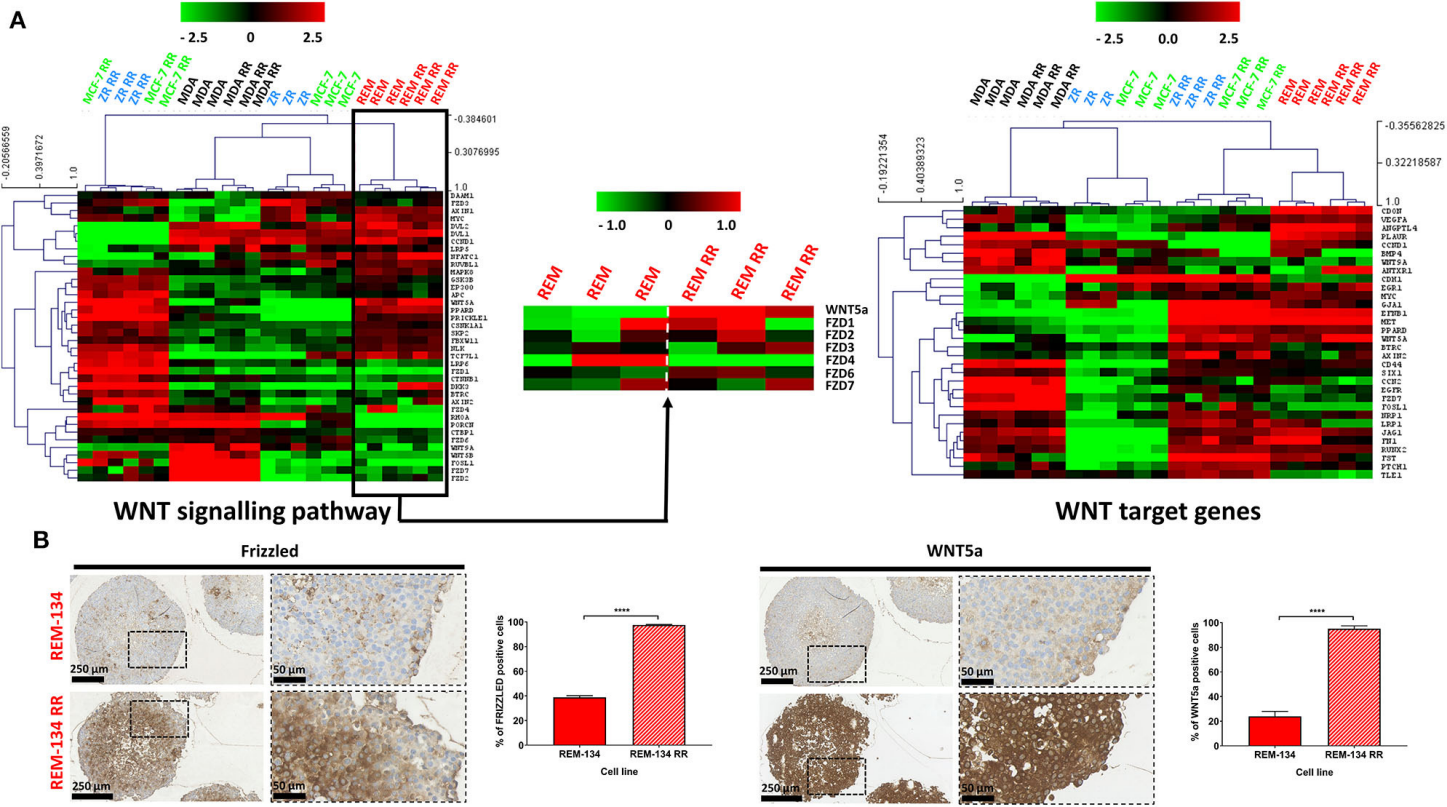
WNT signaling is increased in MCF-7 RR, ZR-751 RR, and REM-134 RR cell lines. **(A)** Heatmap showing log2 mean-centered gene expression profiles between parental and RR cell lines in respect of the WNT signaling pathway (left heatmap) and WNT target genes (right heatmap). WNT5a and represented FRIZZLED genes are shown separately for REM-134 and REM-134 RR cell lines (center heatmap). Genes taken from the KEGG pathway database (55); red = higher expression, black = no change, green = lower expression. The gene lists are shown in Supplementary Table 5. Heatmap clustering was carried out using Pearson correlation with average linkage. **(B)** IHC of pan-Frizzled and WNT5a expression in REM-134 and REM-134 RR MTS with quantitative analysis of the % of positively stained cells (unpaired, two tailed *t*-test; data expressed as mean ± SEM, *n* = 3, ****p ≤ 0.0001).

WNT signaling was studied due to its apparent role in radioresistance and EMT (54); this pathway was investigated using WNT signaling pathway gene expression signatures and WNT signaling downstream targets, both acquired from the KEGG database (55) (genes lists are provided in Supplementary Table 5). The ZR-751 and MCF-7 parental cells were found to have lower expression of WNT pathway and target genes compared to the other cell lines. The MCF-7 RR, ZR-751 RR, REM-134, and REM-134 RR cell lines clustered together with a similar pattern of gene expression consistent with WNT target gene activation. Interestingly, the pattern of expression of WNT signaling pathway members was found to be different between the ER^+^ HCB RR cell lines and both the REM-134 parental and RR lines, potentially suggesting that different WNT signaling pathways in these models may be responsible for the downstream WNT target activation. When the REM-134 and REM-134 RR cell lines were analyzed separately for key differentially expressed (false discovery rate = 0.01) members of the WNT pathway represented in the dataset, clear differences were observed, with the REM-134 RR cell line showing overall higher expression of frizzled family members 1/3/6 and lower expression of frizzled members 2/4, in addition to around 4-fold higher expression of WNT5a (Figure 7A). These results were further investigated using IHC in REM-134 and REM-134 RR MTS. Results showed significantly increased expression of WNT5a and pan-frizzled in the REM-134 RR MTS compared to the parental MTS (Figure 7B). Increased WNT5a expression was also identified in MCF-7 RR and ZR-751 RR MTS in comparison to their respective parental cell lines (Supplementary Figure 4).

### Canine Radioresistant Cell Lines Maintain Their Original Intrinsic Breast Cancer Subtype

**Figure 8:**
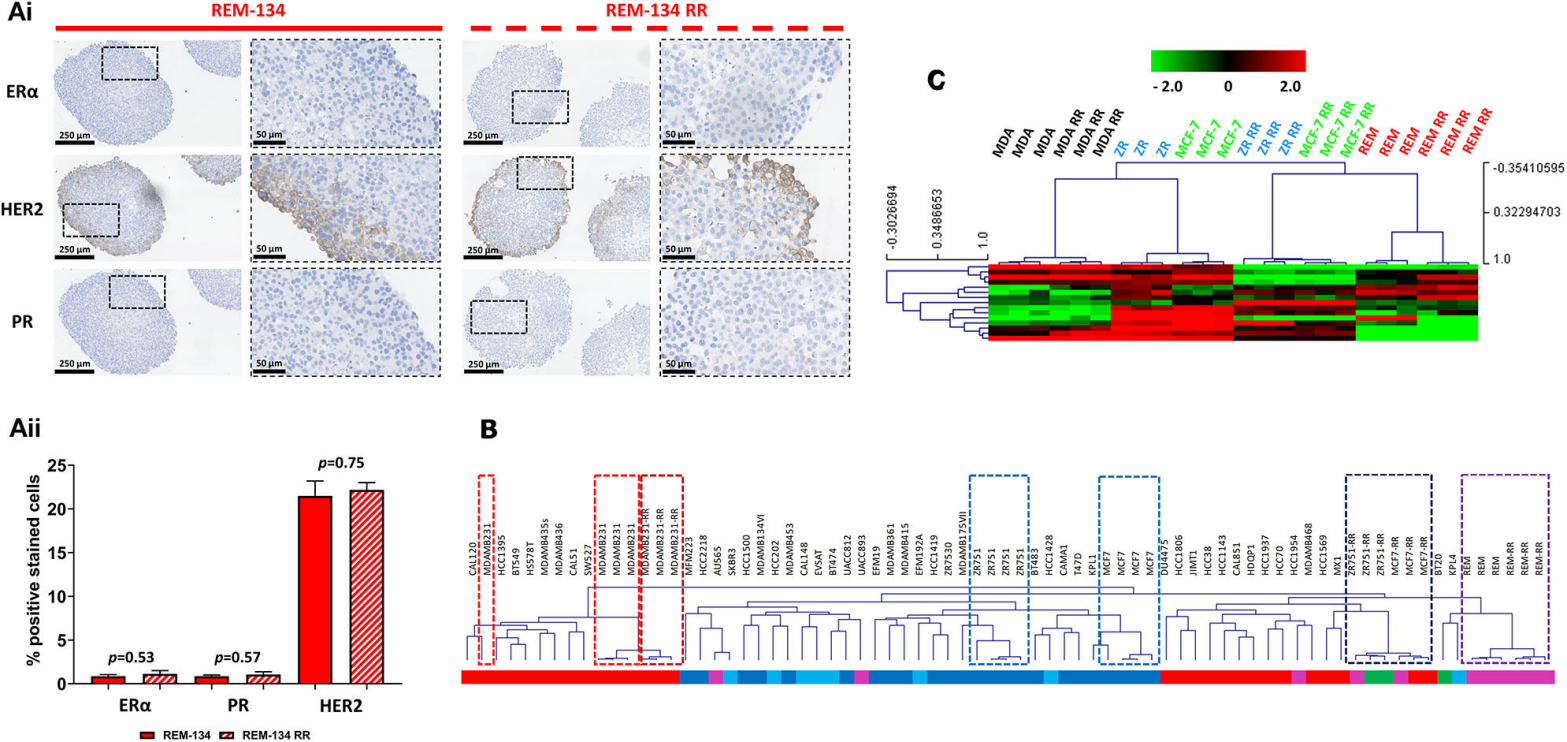
Breast cancer receptor status, ER signaling and intrinsic breast cancer subtype in the canine and human parental and RR cell lines. **(Ai)** IHC of ERα, HER2 and PR expression in REM-134 and REM-134 RR MTS. **(Aii)** Quantitative analysis of the % of ERα, PR and HER2 positively stained cells (unpaired, two tailed *t*-test; data expressed as mean ± SEM, *n* = 3). **(B)** The data generated within this study was integrated with a public gene expression dataset (GSE50811) of 51 breast cancer cell lines. Hierarchical clustering of parental and RR cell lines was based on the Sørlie 2003 intrinsic genes (24); subgroup classification was based on correlation to centroids and was performed using the genefu packaging in R; red = basal, dark blue = luminal A, light blue = luminal B, purple = HER2-overexpressing, green = normal-like. **(C)** Heatmap showing the log2 mean-centered expression profile of a published ER signaling gene signature (56); red = higher expression, black = no change, green = lower expression. Heatmap clustering was carried out using Pearson correlation with average linkage.

Receptor status in the REM-134 and REM-134 RR cell lines was investigated through IHC using MTS. No change in receptor expression was identified with the acquisition of radioresistance, with both the REM-134 and REM-134 RR cell lines classified as ER^−^/PR^−^/HER2^+^ (Figure 8A and Table 2). Although no change was identified in receptor expression between the parental and RR MDA-MB-231 cell lines (both classified as ER^−^/PR^−^/HER2^−^), a difference was seen in the MCF-7 and ZR-751 cell lines. Both MCF-7 and ZR-751 cell lines were classified as ER^+^/PR^+^/HER2^−^; however, their RR derivatives lost ER and PR expression, becoming ER^−^/PR^−^/HER2^−^ (Supplementary Figure 5). Further investigation of cell line classification was performed through integration of the gene expression data from this study with a public gene expression dataset (GSE50811) of 51 breast cancer cell lines. Both the REM-134 and REM-134 RR cell lines clustered tightly and were classified, by correlation to centroids, as belonging to the HER2-overexpressing subtype. They also clustered near the MCF-7 RR and ZR-751 RR cell lines, which were classified as basal/HER2-overexpressing and as normal-like/HER2-overexpressing, respectively. The classification of the MCF-7 RR and ZR-751 RR was different to their parental lines; as anticipated, both of the parental cell lines were classified as luminal A. Predictably, the parental and RR MDA-MB-231 cell lines clustered near each other and were classified as the basal breast cancer subtype (Figure 8B and Table 2).

**Table 2:** Immunohistochemical and molecular subtype classification of the canine and human parental and RR cell lines.

**Cell line**	**Immunohistochemical classification**	**Molecular classification**
MCF-7	ER^+^/PR^+^/HER2^−^	Luminal A
MCF-7 RR	ER^−^/PR^−^/HER2^−^	Basal/HER2-overexpressing
ZR-751	ER^+^/PR^+^/HER2^−^	Luminal A
ZR-751 RR	ER^−^/PR^−^/HER2^−^	Normal-like/HER2-overexpressing
MDA-MB-231	ER^−^/PR^−^/HER2^−^	Basal
MDA-MB-231 RR	ER^−^/PR^−^/HER2^−^	Basal
REM-134	ER^−^/PR^−^/HER2^+^	HER2-overexpressing
REM-134 RR	ER^−^/PR^−^/HER2^+^	HER2-overexpressing

ER signaling was investigated using a published ER signaling gene expression signature (56). As expected, the human ER^+^ parental cell lines (MCF-7 and ZR-751) were characterized by high expression of all of these genes, while in comparison their RR derivatives, together with the REM-134 and REM-134 RR cell lines, were found overall to have lower expression levels of these genes (Figure 8C).

### Canine Parental and Radioresistant Cell Lines Show PI3K and MAPK Activity

**Figure 9:**
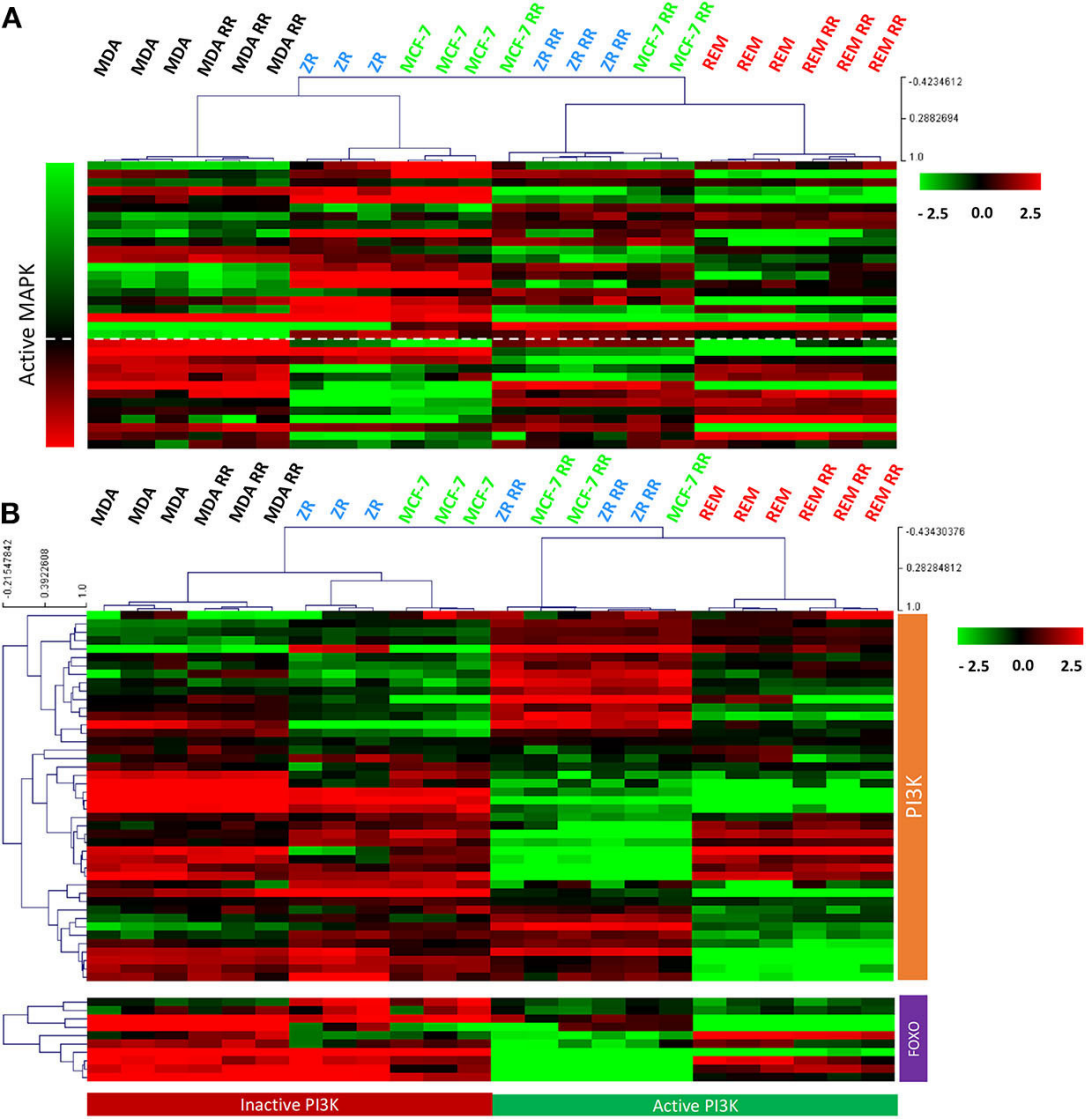
MAPK and PI3K pathway activity in the canine and human parental and RR cell lines. **(A)** Heatmap showing log2 mean-centered gene expression profiles between parental and RR cell lines in respect to a MAPK pathway activity gene signature (57). Heatmap clustering was carried out using Pearson correlation with average linkage, red = higher expression, black = no change, green = lower expression. **(B)** Heatmap showing log2 mean-centered gene expression profiles between parental and RR cell lines in respect of the PI3K pathway [associated genes taken from the PI3K KEGG pathway (upper heatmap) and FOXO-regulated genes (lower heatmap) (55)]. Heatmap clustering was carried out using Pearson correlation with average linkage, red = higher expression, black = no change, green = lower expression.

After we identified that the canine cell lines expressed HER2, signal transduction pathways downstream of the HER/ERBB tyrosine-kinase receptor family were further evaluated. MAPK pathway activity was assessed using a gene expression signature that had previously been published (57). Using a combination of FOXO-regulated genes (which, as downstream targets of inhibition by PI3K, have the opposite pattern of expression to PI3K activity) (58) and genes obtained from the KEGG pathway database (55), PI3K activity was assessed. Results from supervised gene expression analysis were consistent with PI3K and MAPK signaling activity in the REM-134, REM-134 RR, MCF-7 RR and ZR-751 RR cell lines, whereas the MCF-7 and ZR-751 cell lines exhibited inactive PI3K and MAPK activity. Inactive PI3K and active MAPK signaling were also observed in the parental and RR MDA-MB-231 cell lines (Figures 9A,B). Western blot analysis of untreated lysates from all cell lines confirmed the presence of phosphorylated ERK1/2 (MAPK activation) and phosphorylated AKT (PI3K activation) (Supplementary Figure 6).

## Discussion

Radiotherapy is a frequently used curative and palliative treatment for a wide range of human and canine tumors. Unfortunately, intrinsic and acquired radioresistance can significantly limit its efficacy and ultimately leads to local recurrence, disease progression or metastasis. In this study, we developed a canine mammary cancer radioresistant cell line and investigated the cellular mechanisms related to the development of acquired radioresistance. We subsequently performed a comparative analysis of this resistant model with our previously developed HBC radioresistant cell lines, characterizing their inherent differences through genetic, molecular and cell biology approaches.

Intrinsic radiosensitivities of the panel of cell lines was first investigated through CF assays. The REM-134 cell line showed significantly greater radioresistance than that of the human cell lines. Intrinsic radioresistance of the 3 human cell lines was not related to subtype, with the ER^+^ and ER^−^ cell lines showing a similar response to doses of radiation up to 2 Gy. Clinical research studies have identified that HBC subtype is associated with tumor radiosensitivity. One such study investigated invasive breast cancer local recurrence rates following breast-conserving surgery with subsequent adjuvant RT. Their results identified recurrence rates of 0.8% for luminal A, 1.5% for luminal B, 7.1% for basal and 8.4% for HER2-overexpressing HBC subtypes (59). Other studies indicate that triple negative breast cancers and HER2-overexpressing breast cancers that are treated with post-mastectomy RT have increased risks of locoregional recurrence and metastasis, along with significantly reduced overall survival (59–61). However, our results are in accordance with an *in vitro* cell line study which showed that there was no association between HBC subtype and their intrinsic radiosensitivity (62).

Following the 12-week radiation exposure protocol, radioresistance development was verified using CF and SRB proliferation assays. All developed RR cell lines showed greater resistance to a single fractionated radiation dose compared to their parental cells. These results validated their utility as an *in vitro* model system to characterize their resistant phenotype and examine the mechanisms associated with the development of acquired radioresistance. Our protocol was able to generate RR models that exhibited significant differences in CF ability compared to parental cells when exposed to 2 Gy, a standard treatment dose routinely used in HBC patients. This is an important consideration for any resistance development protocol, as differences seen between radiosensitive and radioresistant cells at doses comparable to those used in the clinic will produce more translational data. Our development protocol therefore has significant advantages over others used in the literature, which only managed to generate HBC RR cell lines that had significantly different CF ability compared to their respective parental cells at considerably higher radiation doses (8–10 Gy) (63). Our protocol was also able to produce a radioresistant phenotype that was maintained in REM-134 RR cells that had not received radiation for 6 months. This result is similar to that observed with our previously developed MCF-7 RR cell line (34); this indicates that the acquisition of radioresistance in both our human and canine RR cell line models was not transient. Our results differ from those of other studies which were unable to generate stable HBC radioresistant models (64). These differing results are likely due to the use of different radioresistance development protocols in each study and highlights the need to perform frequent CF and/or SRB proliferation assays to verify maintenance of the radioresistant phenotype.

Following the generation of the REM-134 RR cell line, we evaluated the ability of both the parental and RR cells to generate MTS for use within the study. The HBC MTS used in this study have been previously validated for use as 3D tumor models (34). Using the spinner flask method, we successfully generated MTS from both canine cell lines. This result, to our knowledge, is the first time that REM-134 MTS have been generated. MTS reproduce various aspects of the *in vivo* tumor microenvironment, including low oxygen levels, creating necrotic, peri-necrotic, and hypoxic regions. Proliferative gradients found within cancers are also present in MTS (65). These characteristics were present within the REM-134 MTS produced in this study. Hypoxyprobe-1, a chemical that has been used to detect hypoxic regions in various cancer models (66–68), was used in our MTS model. Hypoxyprobe-1 staining showed that low oxygen regions were present predominantly in the more central areas of MTS, both within and surrounding the necrotic core. Proliferating cells, identified through Ki67 staining, were largely located around the periphery.

Transcriptomic data was initially used to distinguish genes whose expression was significantly changed between the REM-134 and REM-134 RR cell lines following the generation of the RR models. Enriched pathways identified from this analysis, which had previously been linked with radioresistance in studies using human samples, were taken forward for further investigation; these included proliferation, EMT and WNT signaling pathways.

Differences in proliferation between the cell lines were investigated through SRB assays, transcriptomic and IHC analysis. Although the SRB data showed that the ZR-751 RR cell line had an increased rate of proliferation compared to its parental cell line, all of the other analyses, including the gene expression profiles and IHC for the other cell lines, suggested that both the human and canine RR cell lines had reduced proliferation rates. These results are in line with a previous study which identified reduced proliferation rates in human prostate cancer RR cell lines (69). Clinical effects of RT are largely due to the direct and indirect DNA damage it causes. In rapidly-dividing cells there is little time for DNA damage to be repaired by processes such as non-homologous end joining and homologous recombination. If a damaged cell enters cell division with uncorrected DNA damage, then the cell will likely die due to various radiation-induced cell death mechanisms such as mitotic catastrophe, apoptosis, autophagy, necrosis, or senescence. Lower proliferation rates may therefore provide these RR cells with greater time to repair these sites of DNA damage.

Among CMT patients, ~50% of carcinomas will metastasize to local lymph nodes, leading to distant metastases (lung and bone) and death (70, 71). A similar situation occurs in HBC patients; studies indicate that ~7% of patients present with metastatic tumors, while ~20% of patients that are initially diagnosed with local disease subsequently go on to develop metastatic disease (72). Studies that investigate the molecular mechanisms involved in cancer metastasis are therefore of significant clinical importance. In our study, all our RR cell lines showed increased migration and invasion abilities, suggestive of a more aggressive phenotype; this phenotypic change could indicate that RR cells have greater locally invasive and metastatic potentials, factors which are poor prognostic indicators for clinical patients.

Using an initial unsupervised analysis, downregulation of genes involved in HIPPO signaling was identified in the REM-134 RR cell line. This pathway regulates cellular proliferation and apoptosis and has multiple family members that act as tumor suppressors (73). Activation of the HIPPO signaling pathway has also been shown to antagonize WNT signaling, whereas inhibition of HIPPO signaling (causing YAP hypophosphorylation and nuclear localization) can induce EMT (74, 75). Downregulation of HIPPO signaling may therefore be involved in the increase in WNT signaling and EMT seen in the REM-134 RR cell line.

Malignant cellular transformation that leads to loss of epithelial morphology, reduced cellular contact and increased cell migration/invasion is an important feature of EMT (76–78) and is associated with poor prognosis (79). Significant morphological changes were observed in the MCF-7 RR and ZR-751 RR cell lines compared with their parental cells, with the RR cells gaining a more mesenchymal phenotype. The morphology of the MDA-MB-231 and REM-134 cell lines was quite different to that of the parental ER^+^ cell lines, with the former exhibiting a typical mesenchymal phenotype. Although no significant differences were seen in their RR derivatives, their morphology was similar to the MCF-7 RR and ZR-751 RR cell lines. IHC and gene expression analysis were used to investigate these results further. Firstly, using IHC to investigate established EMT breast cancer biomarkers (80), we demonstrated that the MCF-7 RR and ZR-751 RR cell lines exhibited downregulated E-cadherin expression and upregulated vimentin, N-cadherin and SNAIL expression compared to their parental cells. In contrast to these results, the parental and RR MDA-MB-231 cell lines exhibited low E-cadherin and high vimentin expression. Interestingly, the REM-134 and REM-134 RR cell lines showed a hybrid/intermediate epithelial-mesenchymal phenotype, with E-cadherin and vimentin both being expressed. These results were supported by identifying gene expression profiles in our cell lines through the use of a previously published cancer cell EMT-signature (53). Similarities were seen in the MCF-7 RR, ZR-751 RR, REM-134, and REM-134 RR cell lines, which all showed a mixed expression pattern with high expression of mesenchymal and epithelial genes. These results again suggested that these RR cell lines possess a hybrid/transitional phenotype that traverses the epithelial and mesenchymal states (77). Previous studies have suggested that a hybrid EMT state is linked with the presence of stem-like properties, increased cellular plasticity and enhanced migratory/metastatic abilities. It is thought that this state may represent a cellular survival response to stressful environments (81, 82). A multitude of signaling cascades can stimulate EMT in non-cancerous and cancerous cells, including various receptor tyrosine kinase pathways, Wnt-β-catenin and Notch signaling (83, 84). RT can also activate EMT through increasing the expression of TGFβ (85, 86). In our cell lines, WNT signaling was activated in the MCF-7 RR, ZR-751 RR, REM-134, and REM-134 RR cell lines, suggestive of a potential mechanism through which the cells underwent EMT. These results also showed that the acquisition of radioresistance in the human ER^+^ cell lines led to the development of a phenotype similar to that of the canine cell lines.

Previous research studies have investigated the value of CMT as a metastatic model for HBC. Metastatic CMT, in comparison to non-metastasizing tumors, have been shown to exhibit upregulation of genes associated with cell cycle regulation, DNA damage repair, extracellular matrix remodeling, proteasomal degradation and protein folding, while genes involved in cellular differentiation, growth factor signaling and actin organization are downregulated. Of these differentially expressed canine genes, 25% were discovered to be linked to HBC (9). Comparable results to these reported at gene level have also been observed at the intracellular protein level. One study detected 21 proteins (predominantly associated with cell adhesion, extracellular matrix remodeling and hypoxic resistance) that were differentially expressed in canine mammary carcinomas which were classified as either metastasizing or non-metastasizing. The majority (19/21) of these proteins were linked with metastasis or malignancy in a range of human cancers, of which 9 had comparable expression patterns to that seen in HBC patients (87). The partly overlapping transcriptome and proteome of metastatic CMT and HBC indicates that there must be similar pathways/mechanisms involved in breast carcinogenesis and pathogenesis between the two species. These studies also demonstrate that metastatic CMT are an appropriate translational model for metastatic HBC. Similarities between the REM-134 and REM-134 RR transcriptomic data produced in this study and the results from the previous studies discussed here provides further evidence of the value of using these cell line models as a metastatic model of human and canine disease.

HBC is typically graded and characterized through IHC with analysis of expression levels of various receptors such as HER2, ER and PR. Additionally, gene expression profiling has been successfully employed to classify breast tumors into luminal A, luminal B, HER2-overexpressing, basal and normal-like intrinsic subtypes (21, 22). As previously mentioned, these varying subtypes exhibit differing inherent sensitivities to RT, indicate prognosis and can influence which patients receive endocrine and/or targeted therapies (61, 88). To characterize the REM-134 and REM-134 RR cell lines within the context of HBC, we investigated the expression of HER2, ERα, and PR and performed molecular profiling. IHC showed that both parental and RR REM-134 cells expressed HER2, with these cell lines also classifying as HER2-overexpressing through their transcriptional profiles; receptor expression and subtype classification did not change with the acquisition of radioresistance. Similarities again were seen in the RR models produced from ER^+^ cell lines, with a change from luminal A for the MCF-7 and ZR-751 cells to a non-luminal classification for their RR derivatives. The MCF-7 RR cells correlated with the basal/HER2-overexpressing subtypes, while the ZR-751 RR cells correlated with the normal-like/HER2-overexpressing subtypes. Prior to the introduction of HER2-targeted therapies, HER2-overexpressing breast cancers carried a high locoregional recurrence risk and poor overall prognosis (22–24, 89). Luminal A breast cancers typically have an excellent response to RT and endocrine treatments (89); a move away from this subtype classification, as seen in our cell lines, would be consistent with an aggressive, treatment-resistant phenotype. These results indicate that acquired radioresistance can be associated with cellular plasticity, and that gene expression changes can lead to an alteration in molecular subtype. Our results also demonstrate that the RR cells derived from ER^+^ cell lines again show significant similarities to the canine cell lines.

HER2 expression occurs in ~15–30% of all HBC patients (90–92). HER2 protein overexpression has been determined to be both predictive for tumor response to HER2-targeted treatments and prognostic for disease outcome in human patients (93). Several studies have suggested that ~35% of malignant CMT have either HER2 gene or protein expression (94–97) and that HER2 expression is associated with histological grade, proliferation index and tumor size (94, 96, 98). Human and canine genome sequencing has identified significant homology between the HER2 antigens in both species and although further research is needed, results such as these suggest that human-based immunotherapies (e.g., pertuzumab or trastuzumab) or human developed tyrosine kinase inhibitors, could be successfully employed in HER2^+^ CMT (99). The REM-134 cell line and our developed REM-134 RR model would therefore be good *in vitro* models to investigate HER2 signaling in CMT.

In support of the REM-134 HER2-overexpressing classification, we showed that REM-134 cells exhibited lower expression of ER-driven genes in comparison to the human ER^+^ MCF-7 and ZR-751 cell lines. Again, the REM-134 and REM-134 RR cell lines clustered closely with the MCF-7 RR and ZR-751 RR cell lines. In HBC, HER2 expression and ER activity have been shown to have an inverse relationship, with HER2 overexpression being associated with reduced sensitivity to endocrine therapies (100–102). Because of the change in subtype classification and the suggestion of a shift away from ER signaling in the MCF-7 RR and ZR-751 RR cell lines, a situation akin to that seen in the canine cell lines, downstream signaling pathways of the HER/ERBB family were investigated. The phosphatidylinositol-3-kinase (PI3K)/protein kinase B (AKT) pathway is commonly hyperactivated in various cancer types and leads to cellular responses related to survival, proliferation and metabolism (103, 104). RT can also activate the PI3K/AKT pathway, which is associated with intrinsic radioresistance, proliferation, and resistance to hypoxic environments (105, 106). Similarly, activation of the mitogen-activated protein kinase (MAPK) pathway is thought to be a cytoprotective response which can allow cancer cells to repopulate the tumor during fractionated RT (107–111). In this study we found that MCF-7 RR, ZR-751 RR, REM-134, and REM-134 RR gene expression signatures corresponded with activation of the PI3K and MAPK pathways; these pathways may therefore play a significant role in the development of radioresistance in both human and canine patients.

The development of novel RR CMT cell lines opens up the possibility of future *in vivo* studies. Xenograft studies using orthotopic (mammary fat pad) or subcutaneous implantation of paired sensitive and RR REM-134 cells could be used for a multitude of pre-clinical research opportunities. Studies evaluating the effectiveness of radiosensitizing agents, the development of metastatic models or the detection of serum-based biomarkers of radiation response are all achievable with the generation of RR cell lines such as ours.

## Conclusion

This study is the first to report the development and characterization of a novel canine mammary cancer RR cell line which was used as a comparative model for HBC. The generation of new radioresistant models is important, as these will aid the understanding of the molecular mechanisms that drive the development of radioresistance. Similarities in terms of EMT, WNT signaling, estrogen regulation, HER signaling, and subtype classification were identified between the RR cell lines derived from the human ER^+^ cell lines and the canine parental and RR cell lines. These results suggest that the mechanisms involved in the acquisition of radioresistance may be similar in the 2 species. As we continue to appreciate the significant similarities between human and canine mammary tumors, comparative studies will become more important for the investigation of carcinogenesis in both species. We believe that comparative studies of resistant disease will be fundamental for future research, leading to the development of novel treatment strategies that are equally applicable to both human and veterinary patients.

## Data Availability Statement

The datasets presented in this study can be found in online repositories. The names of the repository/repositories and accession number(s) can be found below: https://www.ncbi.nlm.nih.gov/geo/, GSE149988.

## Author Contributions

DA secured funding for this research. MG conceptualized, performed, analyzed, interpreted the laboratory work, wrote the majority of the manuscript, and composed the figures, with significant contributions from AT and JM. AT performed all bioinformatic analysis. CM-P uploaded data to GEO. Critical revisions were made by MG, AT, JM, CM-P, CK, LP, and DA. All authors read and approved the final manuscript.

## Conflict of Interest

The authors declare that the research was conducted in the absence of any commercial or financial relationships that could be construed as a potential conflict of interest.

## Supplementary Material

Supplementary Figure 1 **(A)** Venn diagram showing overlap in gene mappings between human and canine Ensembl gene IDs. **(B)** Violin plot comparing the variance for all matched genes between human and canine datasets. Statistical comparison was performed using two-tailed Mann-Whitney test (non-significant).

Supplementary Figure 2 Log2 mean-centered gene expression profiles of key EMT and Hippo associated genes across all cell lines (selected genes from heatmap in Figure 2). Genes were selected from differential gene expression analysis comparing global gene expression between REM-134 parental and RR cell lines. Heatmap clustering was carried out using Pearson correlation with average linkage based on all differentially expressed genes; red = higher expression, black = no change, green = lower expression.

Supplementary Figure 3 IHC and ICC staining of EMT markers (vimentin, E-cadherin, N-cadherin, and SNAIL) in MCF-7, ZR-751, and MDA-MB-231 parental and RR cell lines.

Supplementary Figure 4 IHC staining for WNT5a in MCF-7 and ZR-751 parental and RR MTS.

Supplementary Figure 5 IHC and ICC staining of hormone receptors (ER, PR, and HER2) in MCF-7, ZR-751, and MDA-MB-231 parental and RR cell lines.

Supplementary Figure 6 **(A)** Combined western blot showing total and phosphorylated ERK1 and ERK2 and total and phosphorylated pan-AKT in parental and RR cell lines. Splicing was performed in the images shown in part (b) to remove redundant lanes to produce this figure. **(B)** Original western blot images from radiation time course experiments in all 8 cell lines. Bands shown in part (a) are highlighted here, which represent untreated control samples.

Supplementary Table 1 RIN values for all samples used in gene expression analysis.

Supplementary Table 2 List of genes differentially expressed between the REM-134 and REM-134 RR cell lines.

Supplementary Table 3 List of G1/S phase regulatory genes taken from the KEGG database cell cycle pathway.

Supplementary Table 4 Epithelial and mesenchymal gene lists from a published EMT signature.

Supplementary Table 5 WNT pathway signaling and target genes taken from the KEGG database.
