# Corrigendum: Comparative Analysis of the Development of Acquired Radioresistance in Canine and Human Mammary Cancer Cell Lines

**DOI:** 10.3389/fvets.2021.664680

**Published:** 2021-03-16

**Authors:** Mark Gray, Arran K. Turnbull, James Meehan, Carlos Martínez-Pérez, Charlene Kay, Lisa Y. Pang, David J. Argyle

**Affiliations:** ^1^The Royal (Dick) School of Veterinary Studies and Roslin Institute, University of Edinburgh, Edinburgh, United Kingdom; ^2^Translational Oncology Research Group, Institute of Genetics and Molecular Medicine, Western General Hospital, University of Edinburgh, Edinburgh, United Kingdom; ^3^Breast Cancer Now Edinburgh Research Team, Institute of Genetics and Molecular Medicine, Western General Hospital, University of Edinburgh, Edinburgh, United Kingdom

**Keywords:** canine breast cancer models, human breast cancer, radioresistance, global gene analysis, characterization of radioresistant cell lines, comparative oncology

In the original article, there was a mistake in the legend for figure 9C as published. Splicing was performed in the image to remove redundant lanes, but this was not originally stated in the figure legend. The band representing ZR-751 t-ERK had also been spliced in wrongly. This error has now been corrected. Figure 9C has now been moved to the supplementary figures and has been uploaded separately. The correct legend appears below.

**Supplementary Figure 6:**
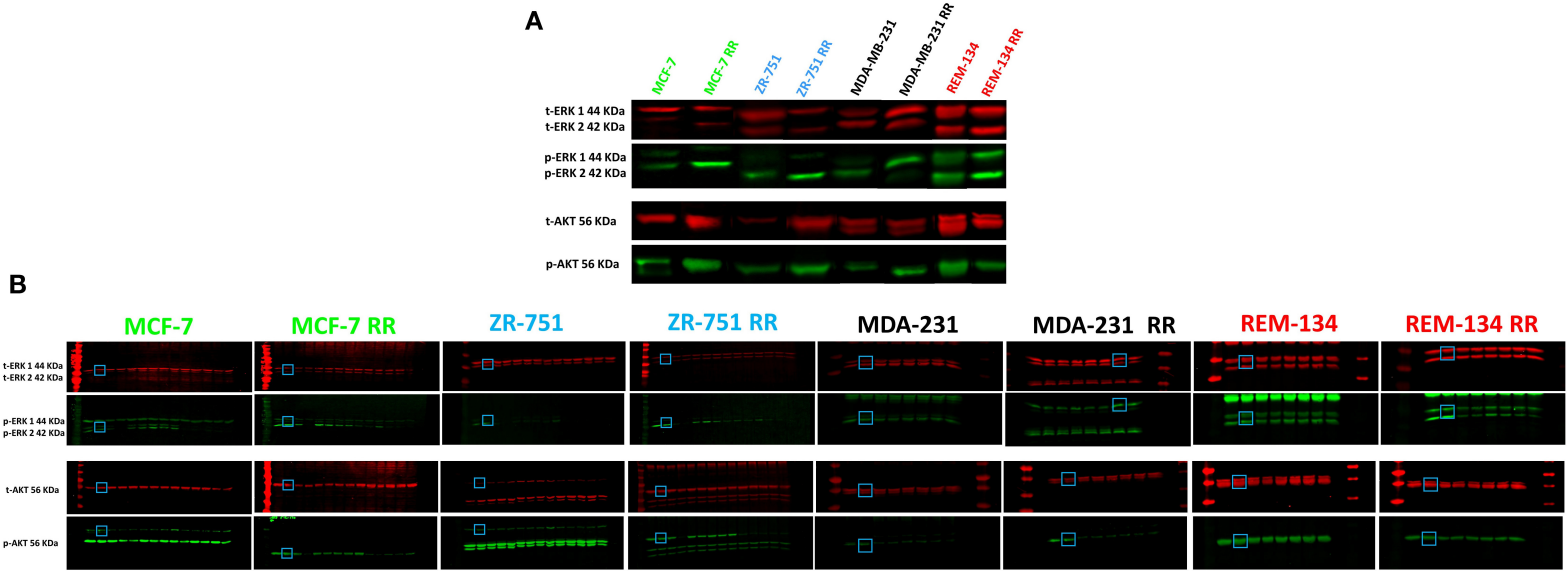
**(A)** Combined western blot showing total and phosphorylated ERK1 and ERK2 and total and phosphorylated pan-AKT in parental and RR cell lines. Splicing was performed in the images shown in part (b) to remove redundant lanes to produce this figure. **(B)** Original western blot images from radiation time course experiments in all 8 cell lines. Bands shown in part (a) are highlighted here, which represent untreated control samples.

The authors apologize for this error and state that this does not change the scientific conclusions of the article in any way. The original article has been updated.

