# Corrigendum: Comparative analysis of the development of acquired radioresistance in canine and human mammary cancer cell lines

**DOI:** 10.3389/fvets.2025.1615706

**Published:** 2025-05-28

**Authors:** Mark Gray, Arran K. Turnbull, James Meehan, Carlos Martínez-Pérez, Charlene Kay, Lisa Y. Pang, David J. Argyle

**Affiliations:** ^1^The Royal (Dick) School of Veterinary Studies and Roslin Institute, University of Edinburgh, Edinburgh, United Kingdom; ^2^Translational Oncology Research Group, Institute of Genetics and Molecular Medicine, Western General Hospital, University of Edinburgh, Edinburgh, United Kingdom; ^3^Breast Cancer Now Edinburgh Research Team, Institute of Genetics and Molecular Medicine, Western General Hospital, University of Edinburgh, Edinburgh, United Kingdom

**Keywords:** canine breast cancer models, human breast cancer, radioresistance, global gene analysis, characterization of radioresistant cell lines, comparative oncology

In the original article, images of spheroids and a scratch assay from a previous publication from our group in the journal Radiation Oncology were re-used (publication: https://ro-journal.biomedcentral.com/articles/10.1186/s13014-019-1268-2). Whilst this related publication was referenced in the manuscript, the re-use of the figures in their legends was mistakenly not acknowledged. The authors have confirmed that they retain the rights for re-publication/re-use of the images included in the Radiation Oncology publication. The figures in question are 4Ci (images of the MCF-7 and ZR-751 radiosensitive and radioresistant spheroids) and 6A (image of the MDA-MB-231 radioresistant scratch assay). The amended legends appear below.

The caption of Figure 4 previously stated:

**Figure 4**. Radioresistant cell lines have modified basal proliferation rates relative to their parental cells. **(A)** SRB assays showing differences in proliferation rates between MCF-7, ZR-751, MDA-MB-231, and REM-134 cell lines and their derived RR cell lines grown in 2D cultures (2-way ANOVA with Holm-Šídák multiple comparisons test; data expressed as mean ± SEM, *n* = 3, ^****^p ≤ 0.0001; ^***^p ≤ 0.001). **(B)** Heatmap showing log2 mean-centered gene expression profiles of proliferation genes in parental and RR cell lines showing key G1/S phase regulators taken from the KEGG database cell cycle pathway (55); red = higher expression, black = no change, green = lower expression. Heatmap clustering was carried out using Pearson correlation with average linkage. The gene list is shown in **Supplementary Table 3**. **(Ci)** IHC of MTS stained for Ki67 using MCF-7, ZR-751, and REM-134 parental and RR cell lines. **(Cii)** Quantitative analysis of the % of cells with Ki67 staining (unpaired, two tailed *t-*test; data expressed as mean ± SEM, *n* = 3, ^****^p ≤ 0.0001).

The caption has been corrected to:

**Figure 4**. Radioresistant cell lines have modified basal proliferation rates relative to their parental cells. **(A)** SRB assays showing differences in proliferation rates between MCF-7, ZR-751, MDA-MB-231, and REM-134 cell lines and their derived RR cell lines grown in 2D cultures (2-way ANOVA with Holm-Šídák multiple comparisons test; data expressed as mean ± SEM, *n* = 3, ^****^p ≤ 0.0001; ^***^p ≤ 0.001). **(B)** Heatmap showing log2 mean-centered gene expression profiles of proliferation genes in parental and RR cell lines showing key G1/S phase regulators taken from the KEGG database cell cycle pathway (55); red = higher expression, black = no change, green = lower expression. Heatmap clustering was carried out using Pearson correlation with average linkage. The gene list is shown in **Supplementary Table 3**. **(Ci)** IHC of MTS stained for Ki67 using MCF-7, ZR-751 [images reproduced from (34)], and REM-134 parental and RR cell lines. **(Cii)** Quantitative analysis of the % of cells with Ki67 staining (unpaired, two tailed *t*-test; data expressed as mean ± SEM, *n* = 3, ^****^p ≤ 0.0001).

The caption of Figure 6 previously stated:

**Figure 6**. Radioresistant cell lines have increased migration and invasion potential. **(A)** Images of 2D migration and 3D MTS invasion assays comparing the parental and the derived RR cell lines. **(B)** Graphs exhibiting the migration **(Bi)** and invasion assay **(Bii)** results. For the migration assays the relative migratory distance was calculated at each time point up to 48 h and expressed as a % area devoid of cells based on the initial scratched area at day 0. Invasion was assessed up to 96 h post-seeding. Area of MTS at each time point was calculated and expressed as a % of initial MTS area at day 0 (2-way ANOVA with Holm-Šídák multiple comparisons test; data expressed as mean ± SEM, *n* = 3, ^****^p ≤ 0.0001; ^***^p ≤ 0.001; ^*^p ≤ 0.05.

The caption has been corrected to:

**Figure 6**. Radioresistant cell lines have increased migration and invasion potential. **(A)** Images of 2D migration and 3D MTS invasion assays comparing the parental and the derived RR cell lines [MDA-MB-231 image reproduced from (34)]. **(B)** Graphs exhibiting the migration **(Bi)** and invasion assay **(Bii)** results. For the migration assays the relative migratory distance was calculated at each time point up to 48 h and expressed as a % area devoid of cells based on the initial scratched area at day 0. Invasion was assessed up to 96 h post-seeding. Area of MTS at each time point was calculated and expressed as a % of initial MTS area at day 0 (2-way ANOVA with Holm-Šídák multiple comparisons test; data expressed as mean ± SEM, *n* = 3, ^****^p ≤ 0.0001; ^***^p ≤ 0.001; ^*^p ≤ 0.05).

The authors apologize for this error and state that this does not change the scientific conclusions of the article in any way. The original article has been updated.

