# Supplementary figures and images for: Comparative Analysis of the Development of Acquired Radioresistance in Canine and Human Mammary Cancer Cell Lines
Source: Front Vet Sci. 2020 Jul 23;7:439. doi: 10.3389/fvets.2020.00439 (PMC7396503; doi:10.3389/fvets.2020.00439)

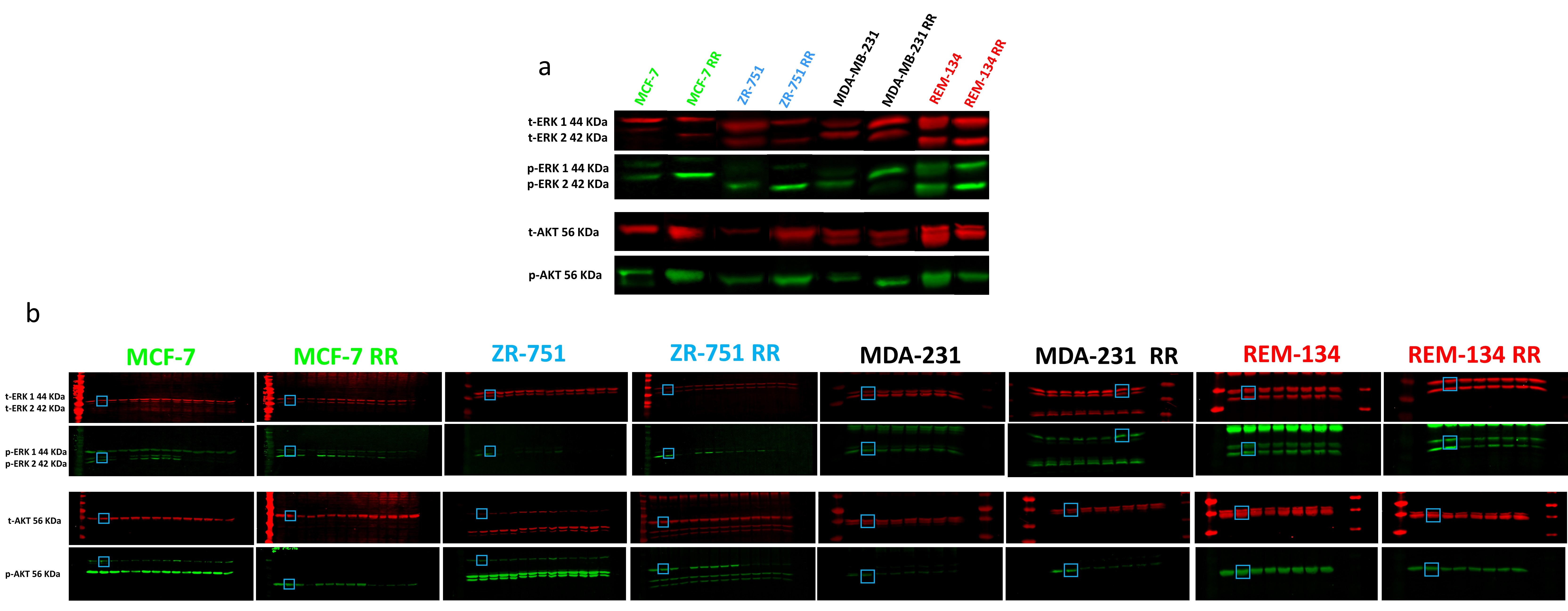

Supplement: Supplementary Figure 1 — (A) Venn diagram showing overlap in gene mappings between human and canine Ensembl gene IDs. (B) Violin plot comparing the variance for all matched genes between human and canine datasets. Statistical comparison was performed using two-tailed Mann-Whitney test (non-significant). [file Image_1.jpg]

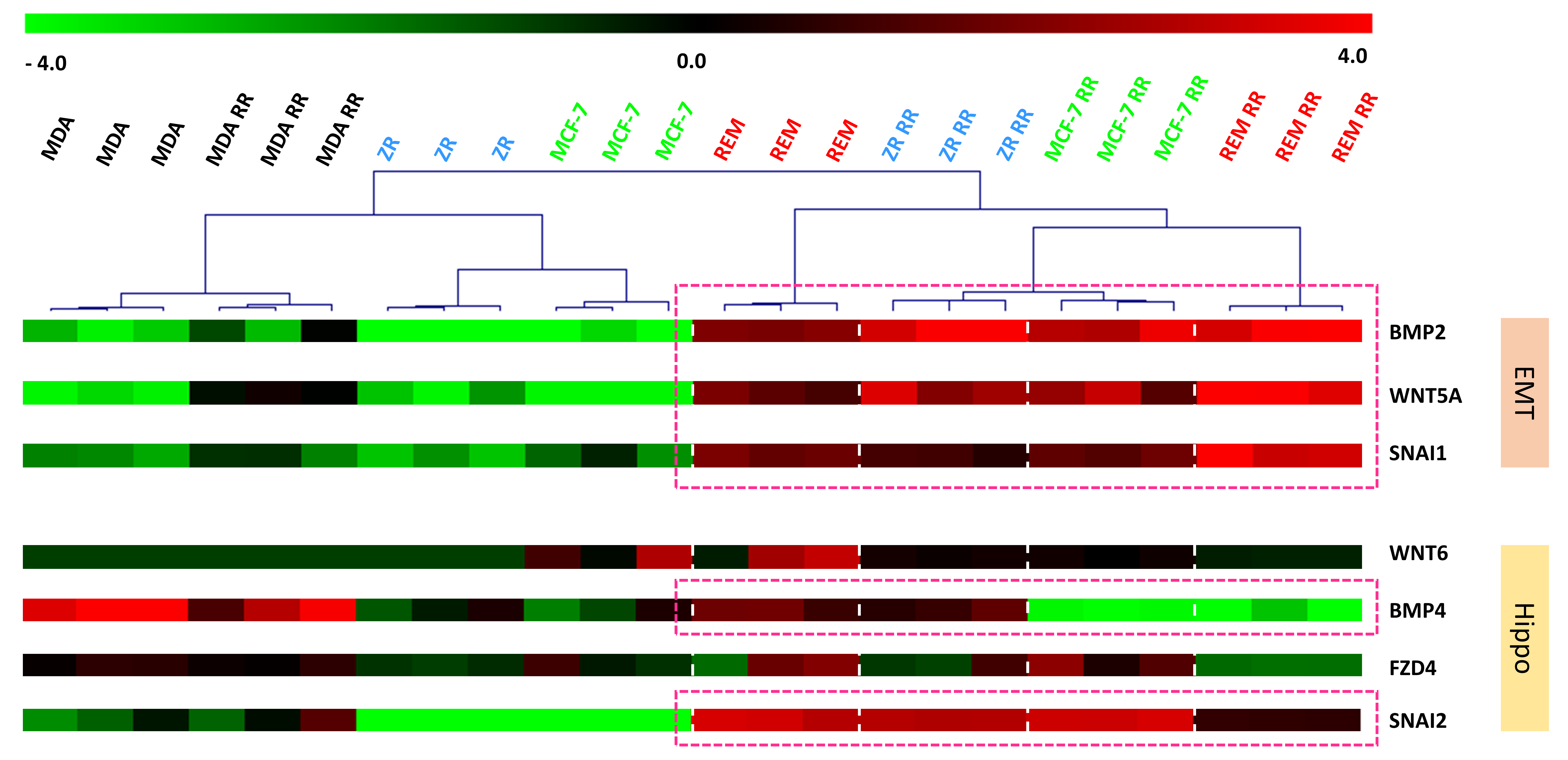

Supplement: Supplementary Figure 2 — Log2 mean-centered gene expression profiles of key EMT and Hippo associated genes across all cell lines (selected genes from heatmap in Figure 2). Genes were selected from differential gene expression analysis comparing global gene expression between REM-134 parental and RR cell lines. Heatmap clustering was carried out using Pearson correlation with average linkage based on all differentially expressed genes; red = higher expression, black = no change, green = lower expression. [file Image_2.TIF]

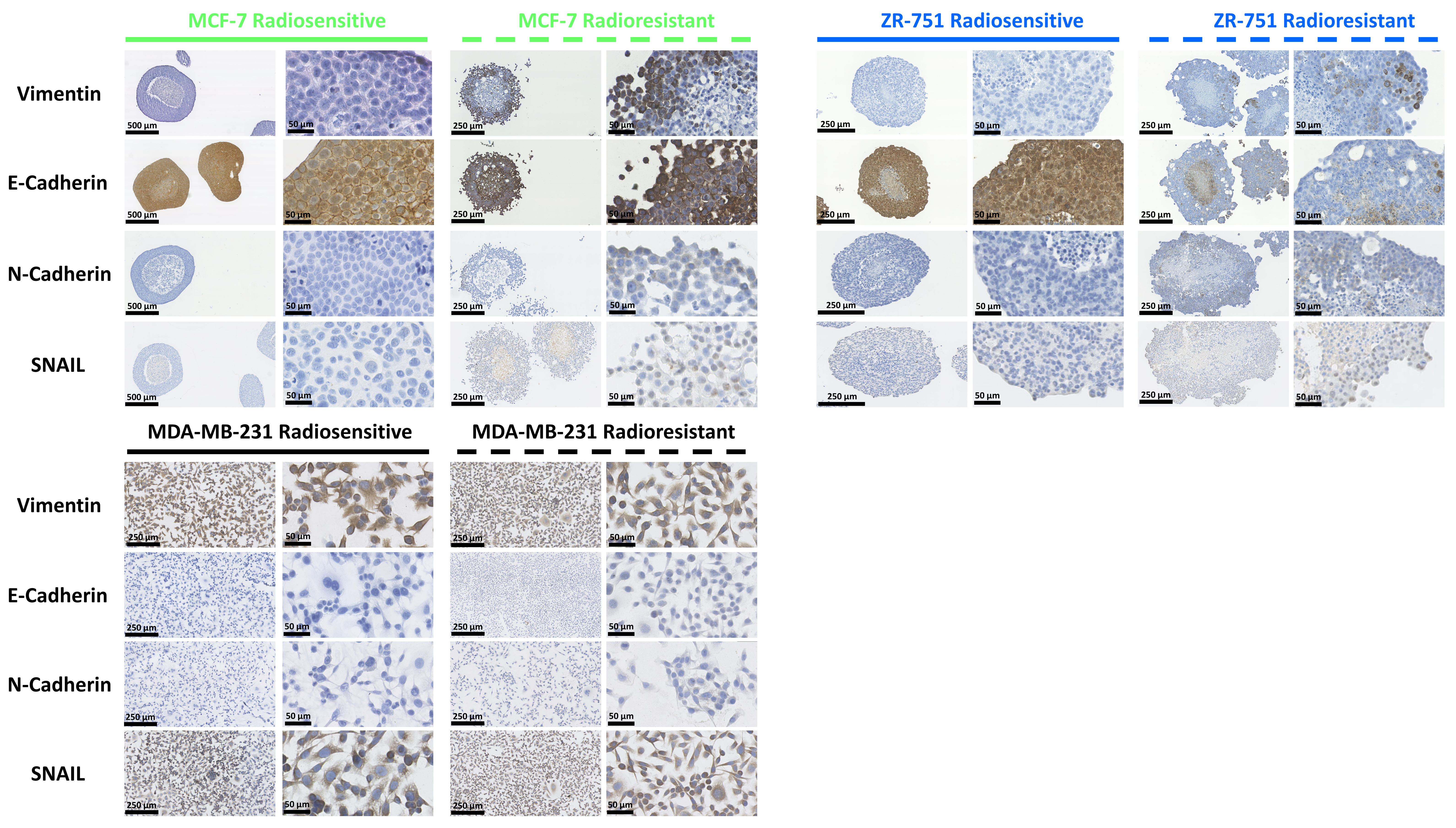

Supplement: Supplementary Figure 3 — IHC and ICC staining of EMT markers (vimentin, E-cadherin, N-cadherin, and SNAIL) in MCF-7, ZR-751, and MDA-MB-231 parental and RR cell lines. [file Image_3.TIF]

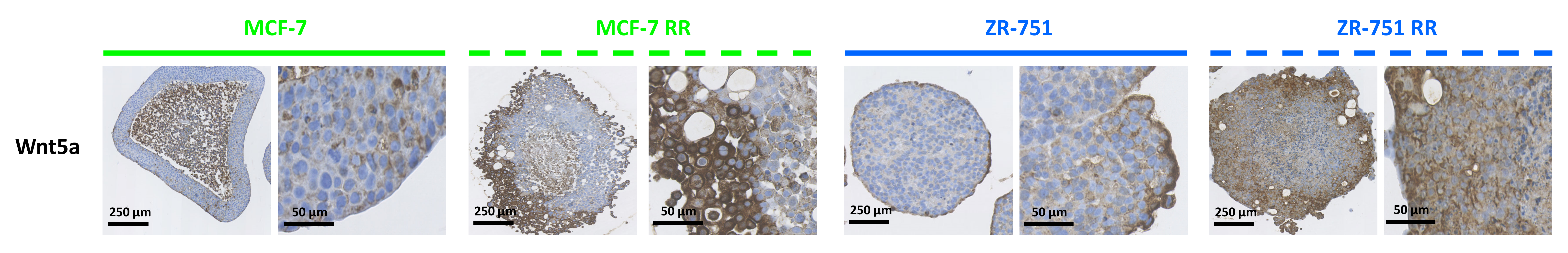

Supplement: Supplementary Figure 4 — IHC staining for WNT5a in MCF-7 and ZR-751 parental and RR MTS. [file Image_4.TIF]

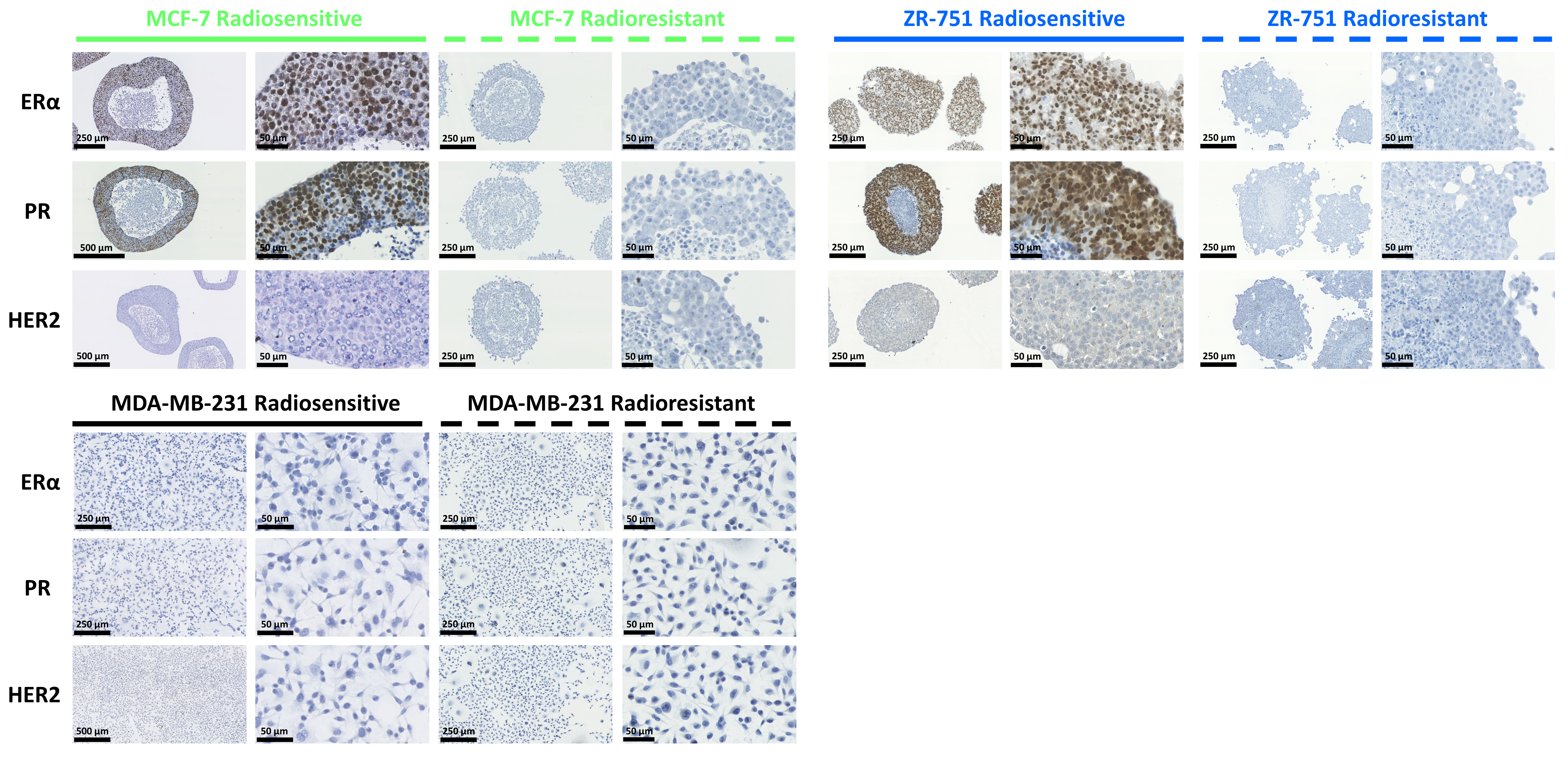

Supplement: Supplementary Figure 5 — IHC and ICC staining of hormone receptors (ER, PR, and HER2) in MCF-7, ZR-751, and MDA-MB-231 parental and RR cell lines. [file Image_5.TIF]

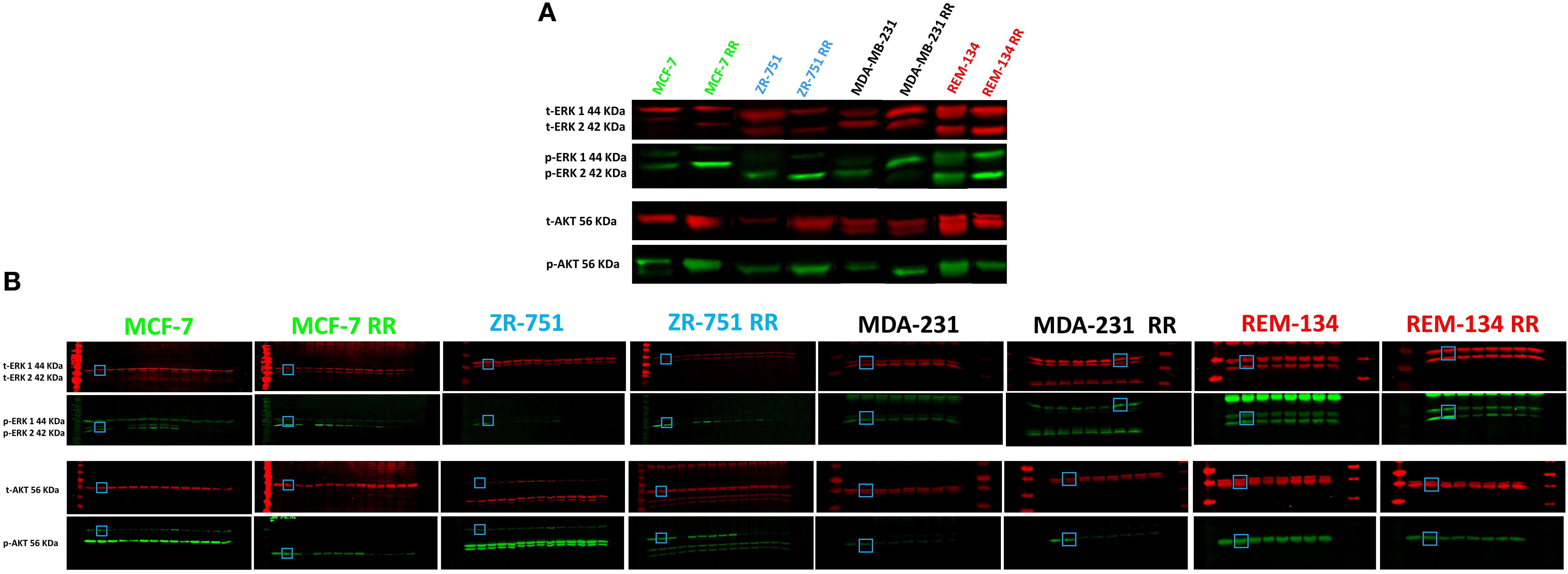

Supplement: Supplementary Figure 6 — (A) Combined western blot showing total and phosphorylated ERK1 and ERK2 and total and phosphorylated pan-AKT in parental and RR cell lines. Splicing was performed in the images shown in part (b) to remove redundant lanes to produce this figure. (B) Original western blot images from radiation time course experiments in all 8 cell lines. Bands shown in part (a) are highlighted here, which represent untreated control samples. [file Image_6.jpg]
